# Factors of influence in prisoner’s dilemma task: a review of medical literature

**DOI:** 10.7717/peerj.12829

**Published:** 2022-01-28

**Authors:** Vasileios Mantas, Artemios Pehlivanidis, Vasileia Kotoula, Katerina Papanikolaou, Georgia Vassiliou, Anthoula Papaiakovou, Charalambos Papageorgiou

**Affiliations:** 1 1st Department of Psychiatry, Eginition Hospital, National and Kapodistrian University of Athens, Athens, Greece; 2 Experimental Therapeutics and Pathophysiology Branch, National Institute of Mental Health, Bathesda, MD, USA; 3 Department of Child Psychiatry, Agia Sophia Children’s Hospital, National and Kapodistrian University of Athens, Athens, Greece

**Keywords:** Prisoner’s dilemma, Medical research, PD

## Abstract

The Prisoner’s Dilemma (PD) is one of the most popular concepts amongst the scientific literature. The task is used in order to study different types of social interactions by giving participants the choice to defect or cooperate in a specific social setting/dilemma. This review focuses on the technical characteristics of the PD task as it is used in medical literature and describes how the different PD settings could influence the players’ behaviour. We identify all the studies that have used the PD task in medical research with human participants and distinguish, following a heuristic approach, seven parameters that can differentiate a PD task, namely (a) the opponent parties’ composition; (b) the type of the opponent as perceived by the players; (c) the interaction flow of the game; (d) the number of rounds; (e) the instructions narrative and options that are given to players; (f) the strategy and (g) the reward matrix and payoffs of the game. We describe how each parameter could influence the final outcome of the PD task and highlight the great variability concerning the settings of these parameters in medical research. Our aim is to point out the heterogeneity of such methods in the past literature and to assist future researchers with their methodology design.

## Introduction

Two criminals are arrested without sufficient evidence. With no means of communicating with one another, the police offers each one a bargain for diminished sentence time. Each prisoner has two options, to ‘betray’ the other by testifying he committed the crime, or to ‘cooperate’ by remaining silent. If one prisoner chooses to betray the other, while the other cooperates, the first will be set free (Temptation payoff—T) while the other serves the maximum sentence (Sucker’s payoff—S). If both prisoners choose to betray each other, they each serve less time (Punishment payoff—P) in prison. If both prisoners cooperate, they will serve the minimum sentence (Reward payoff—R).

The Prisoner’s Dilemma (PD), as described above, was originally devised by Merrill Flood and Melvin Dresher but received its name and final form by Albert W. Tucker. It belongs to the Game Theory family of games and its uniqueness derives from the fact that it represents the conflict between the optimum distribution of rewards (Pareto optimal) and the optimum choice for an individual (Nash equilibrium) (Apt & Grädel, 2011). From the player’s perspective, this corresponds to the conflict of interest between the options of cooperation and defection. The largest payoff for a player occurs with defection while their partner cooperates, mutual cooperation brings a modest payoff to both players and mutual defection yields a lesser amount to each of them. The uniqueness of this dilemma is considered analogous to real life conditions and has helped establish the PD game as a standard and valuable tool for the study of social decision making (Poundstone, 1993). As such, the task has gained great popularity amongst the totality of scientific research. Concerning the medical literature and according to the PubMed database, more than one thousand articles that use the PD task have been published dating as early as 1963.

The present review article focuses on the technical aspects of the PD as this is used in the medical research, where human subjects are involved. In medical literature the PD game has been used to study social behaviour and interaction in healthy populations as well as in cases of pathology including psychiatric disorders. It has also been used in conjunction with neuroimaging techniques such as fMRI (Eimontaite et al., 2019; Thompson et al., 2021) and EEG (Babiloni et al., 2007; Back et al., 2009) to help identify the brain areas and networks that are activated when social decisions are made and to examine whether the activations of these brain areas differ in various populations. Depending on the aims of the study, several parameters need to be considered when setting up a PD experiment. Each of these parameters could impact the outcome of the experiment and thus their selection is a critical step in PD research.

The aim of this review, is to focus on the technical characteristics of the PD as this has been used in medical research involving human participants, in order to identify the parameters that could influence the outcome of the PD task and present the different settings that these parameters could take. Based primarily on the literature but also our own experience in setting up a study that utilised the PD task, we have identified seven parameters that should be considered during the experimental design of the PD. These parameters include: (a) the opponent parties’ composition; (b) the type of the opponent as perceived by the players; (c) the interaction flow of the game; (d) the number of rounds; (e) the instructions, narrative and options that are given to players; (f) the strategy and (g) the reward matrix and payoffs of the game. For each parameter, we present the different settings that are available and when possible which aspects of social behaviour and interaction they would promote. In addition to highlighting the heterogeneity in PD medical research, this review could help researchers to optimise their experimental design in order to best target specific populations and/or research questions.

## Methods

**Figure 1:**
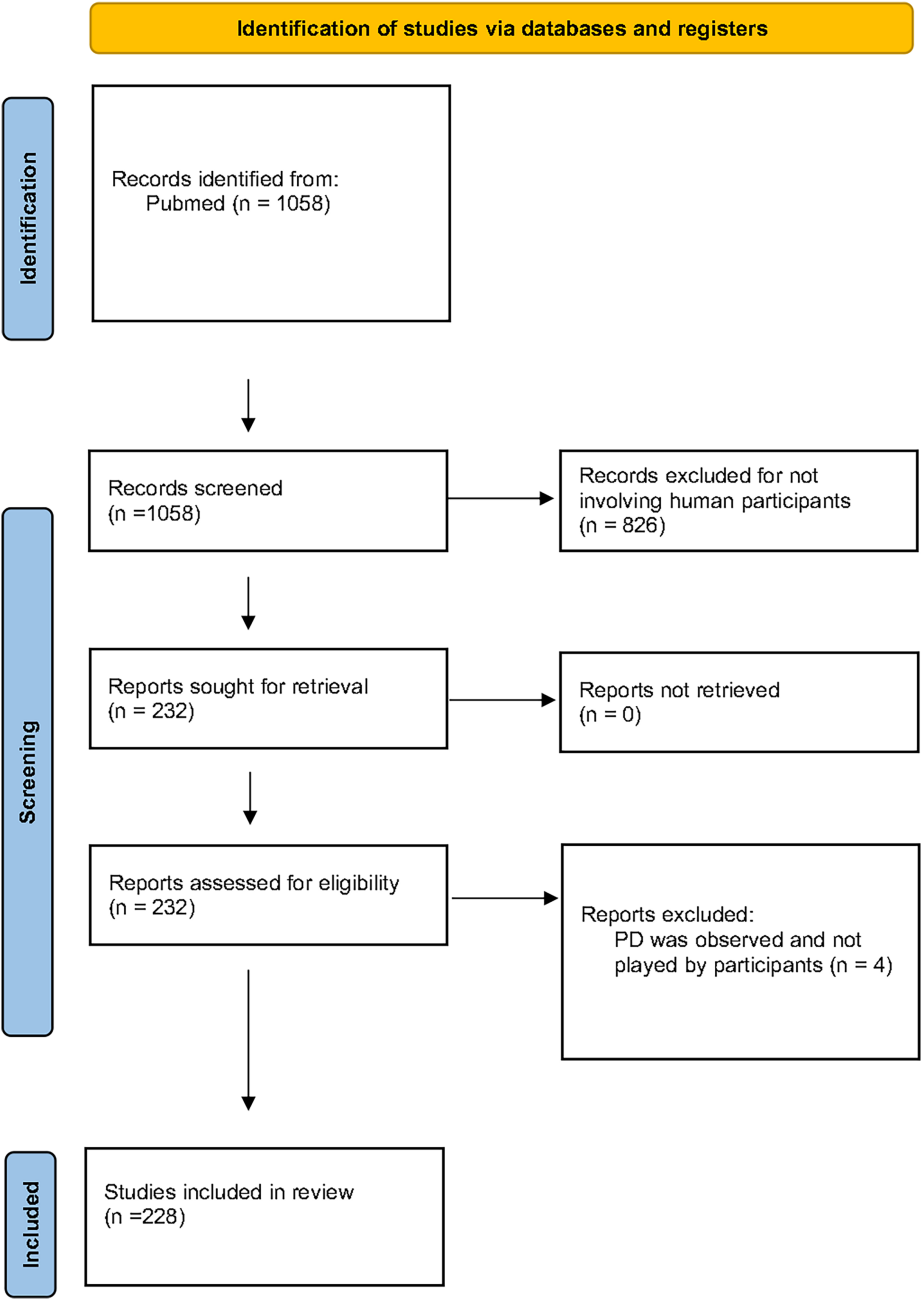
The PRISMA flow diagram. For the purposes of this review we focused on medical literature studies with actual participants. A total of 228 articles are included. We have identified seven parameters, that based on our experience as well as the current literature could significantly differentiate the outcome of a PD experiment. Each article was examined and classified based on the choice that the experimenters have made concerning these seven PD parameters.

The PRISMA guidelines (Fig. 1) were followed in order to identify medical research studies that have used the PD paradigm in human participants. We conducted a search in the PubMed database using the terms ‘(“prisoner’s dilemma”) OR (“prisoner dilemma”) OR (“prisoners dilemma”)’ as keywords. Our research returned 1,058 articles, dated until January 2021. Since our focus was on medical research that has used human participants in the PD experiments, we excluded all the studies that have used computers to simulate human behaviour as well as all studies involving animal research. According to this, 826 articles were excluded. From the 232 remaining publications, four articles were excluded as their methodology involved use of the PD as a paradigm for the subjects to observe or evaluate but not participate. A total of 228 articles were included in this review and were independently evaluated by the first author and three of the co-authors mainly focusing on the PD methodology. The relevant literature references of the 228 articles were also checked by the authors in order to ensure that all studies fulfilling our criteria were included in the review and were not missed during our PubMed research. No new studies were identified by checking the references of the 228 articles.

Based on the literature as well as our personal experience concerning the setup requirements of a PD task, we identified the seven parameters that could significantly differentiate the outcome of a PD experiment. These parameters include: (1) the composition of the opponent parties of the PD game—Opponent parties composition; (2) the type of opponent as perceived by the PD players—Opponent perceived type; (3) the sequence of events during the PD game—Interaction Flow; (4) the number of rounds in the game—Number of Rounds; (5) the Instructions, narrative and options; (6) the Strategy and (7) the Reward matrix and payoffs. Each article was examined and the settings for each of the seven parameters were extracted and can be found in the Supplemental Material. In the ‘Results’ section that follows we define each of these parameters and based on the literature that we reviewed we present the different choices that are available for each parameter and how they could influence the experiment’s outcome. Some indicative studies that have utilized some of the available parameter settings are also presented and discussed.

## Results

### PD parameters

#### Opponent parties’ composition

Opponent parties’ composition refers to the synthesis of the competing PD parties. We use the term “parties” to include PD experiments where the opponents are individuals as well as groups of individuals. Most commonly the PD game is used as a research analogue of social interactions. Such interactions mainly occur between individuals or groups, which constitute the opponent parties. The main difference between individual and group interactions is that in the between-groups condition, members of each group have to settle to an agreement about their representative PD choice.

The vast majority of the PD literature concerns interactions between individuals. There are however, three additional types of PD experiments where interactions cannot be strictly categorized as group or individual interactions. The first of these additional PD types, places the individual in a network of others, where their every PD choice simultaneously determines the outcome of multiple PD interactions between them and their neighbours. This type of PD is mainly used to investigate the dynamics of social networks, for example how the members’ psychopathic traits could influence the group or how dynamic partner updating would influence cooperation levels (Wang, Suri & Watts, 2012). In the second type (IPD-MD: Intergroup Prisoner’s Dilemma-Maximum Difference) (Halevy, Bornstein & Sagiv, 2008), the individual is a member of a group and their PD decision affects both members of their and other conflicting groups. This type of PD has been used in research to investigate specific characteristics of the players behaviour such as the motives behind altruism, including the desire to help one’s group or harm other groups (De Dreu, Dussel & Velden, 2015; Weisel & Böhm, 2015). The third type is that of a multiplayer PD where both the individuals’ and the groups’ rewards depend on the combination of the members’ decisions without involving any direct interaction between individuals. This PD type is a tool for investigating altruistic behaviour and cooperation as it highlights the dilemma between personal and social benefits (Sheldon, Sheldon & Osbaldiston, 2000; Locey, Safin & Rachlin, 2012).

From the research articles included in this review, the majority used interactions between individuals (207 experiments) with fewer studies looking at interactions between groups (10 experiments).

#### Opponent perceived type

Opponent perceived type refers to the players’ beliefs about the nature of their opponents. The subject’s perception of their opponent’s nature is of great importance for the PD game methodology. This is mainly due to the contrasting natures of the PD and that of modern methodology. The PD paradigm is mainly used to investigate the characteristics of social behaviour which per definition requires human interactions, modern methodological environments however, are machine-based and aim to minimize human involvement.

In order to compromise the two, players have to either (a) truly believe that machines could accurately mimic or simulate all aspects of human behaviour and assign to them innate human characteristics like compassion or (b) believe that their opponent is an actual human. This becomes even more complicated when taking into account the fact that machines are often used as a proxy for individual interactions. As a result, when someone confronts a soulless screen, they can never be sure about what it reflects/represents. For that purpose, a common practice is to ask participants, at the end of the experiment, what they believed about their opponent’s nature.

We have identified two categories for the opponent perceived type parameter: human agents playing as experimental subjects and agents with predefined strategy or answers. The first opponent type, human *vs*. human, is a more convenient and realistic model when we intend to study the interaction itself while the second opponent type, human *vs*. agent with predefined strategy, is a more controlled setting and allows the study of individual subject characteristics as well as the comparison between subjects. The latter category could be further separated in order to distinguish whether or not the subject was falsely informed about the human nature of their opponent or manipulated into thinking so. This manipulation is usually achieved by the ‘supposed’ presentation (*i.e*., introduction or photo) or actual presence of a human opponent, signs of communications (*i.e*., messages) and signs of human behaviour (*i.e*., delays). Whether or not this is truly important, it remains controversial. On one hand, Miwa & Terai (2012) have shown that participant’s behaviour during the PD game is influenced by the instructions given about the partner’s nature while Knight & Kagan (1977) stated that playing against an imaginary opponent strongly correlates with decision-making when an opponent is actually present. On the other hand, it has been shown that humans activate brain areas related to mentalizing when they knowingly face computers as co-players (Rilling et al., 2004a; Krach et al., 2008; Kircher et al., 2009). Finally, interaction (cooperation) significantly changes when comparing human and computer opponents, only when communication between the opponents is avoided (Kiesler, Sproull & Waters, 1996).

Another factor to consider when the ‘opponent type’ parameter is concerned is the sex balance of the players or opposing parties which could influence the game decisions (Rapoport & Chammah, 1965). Male opponents usually appear more cooperative than women in same-sex interactions while women cooperate more than men in mixed-sex settings (Rapoport & Chammah, 1965; Balliet et al., 2011; Colman, Pulford & Krockow, 2018). Social proximity is another important factor. Rewards to socially closer persons are valued more than equivalent and or identical rewards to socially distant ones, which in turn alters subjects’ behaviour as the cost of cooperation is compared to its socially discounted benefit (Locey & Rachlin, 2011; Locey, Safin & Rachlin, 2012; Safin, Locey & Rachlin, 2013; Safin, Arfer & Rachlin, 2015). A possible explanation for this, is perceived similarity which signals some degree of kinship (Park & Schaller, 2005), trustworthiness (Koch et al., 2020), likability (Alves, Koch & Unkelbach, 2017, 2018), and opportunity to form a group (Hehman, Flake & Freeman, 2018) and could thus explain cooperation in social dilemmas (Rand & Nowak, 2013).

Only 40% of the PD experiments, included in this review involved interactions between real human subjects. From these, only a few involved a vis-a-vis setting (mostly EEG experiments). The majority of the studies have used computers to conduct interactions (117 experiments). From the 123 PD experiments that have used algorithms, in at least 97 participants were manipulated to believe they were interacting with real humans.

#### Interaction flow

Interaction flow describes the order in which the players take turns throughout the PD game. Based on the interaction flow parameter, PD experiments can be distinguished into ‘simultaneous’ and ‘sequential’. In simultaneous PD, which is the most common type of PD, both parties choose their actions without knowing each others current decision. In the sequential PD, one party makes the first move (Player A) and the other the subsequent move (Player B). In this case, Player B has the benefit of already knowing Player’s A choice before deciding upon their move and thus Player B has a strategic advantage over Player A who is aware of this. Kiyonari, Tanida & Yamagishi (2000) has shown that the cooperation levels in the sequential PD are higher when compared to the simultaneous PD. Locey & Rachlin (2011) implemented a modified version of the sequential type where each participant has the role of both Player A and Player B, as each decision is the answer to the opponent’s last choice and at the same time the first move in the new round.

Using the interaction flow parameter, PD experiments can also be distinguished based on the total number (sum) of rounds. When only one round is involved the game is called ‘one-shot’, else, the game is called ‘iterated’. There is a great difference between the two types, as in ‘one-shot’ PD, the player does not have any information/knowledge concerning their opponent’s strategy that would be based on any previous behaviour—nor the opportunity to be strategic. In this case, defection seems to be the most rational choice. Experiments involving human subjects however, have proven that this is far from true since individuals often choose cooperation in ‘one shot’ trials (Shafir & Tversky, 1992; Wedekind & Milinski, 1996; Wong & Hong, 2005). This justifies the use of ‘one-shot’ PD as a means to evaluate the altruistic nature of the agents. In contrast to ‘one-shot’ PD, ‘iterated’ PD, encompasses the essential time frame for strategy to be assessed and implemented and thus it is more suitable for studying the specifics of the evolution of cooperation (*i.e*., reputation, hierarchy, strategy). Another form of ‘iterated’ PD is that involving multiple ‘one-shot’ games (Yang & Yue, 2019). In this case, the possibility of a future rematch with the same opponent can induce higher cooperation levels (Bó, 2005; Duffy & Ochs, 2009; Arechar, Kouchaki & Rand, 2018; Rand & Nowak, 2013).

From the articles included in our review, only 29 papers involve a sequential type of PD while 186 involve a simultaneous one.

#### Number of rounds

The ‘number of rounds’ parameter determines the length of the PD game and it is relevant only for the ‘iterated’ type of PD as ‘one-shot’ PD consists of only one round. Our literature search revealed a great variability in the number of rounds used in research, with most studies using 10 to 30 rounds. The total number of rounds in a PD experiment, is considered important for achieving cooperation. The fewer the rounds, the bigger the risk of unsuccessful negotiation and loss. Lave (1965) suggests a minimum number of 3 * (P − S)/(R − P ) rounds, where R stands for reward, P for punishment and S is for sucker payoff (see introduction and ‘reward matrix and payoffs’ section) to achieve mutual cooperation based on the reward matrix and Chang et al. (2010) demonstrate that 12 rounds are sufficient for individuals to achieve stable levels of cooperation.

As a PD game approaches to the end, the number of remaining rounds decreases and this can influence the participants’ behaviour; end-game effect. As the margin for retaliation is getting smaller, temptation for opportunistic behaviour increases along with the uncertainty concerning the opponent’s attitude towards this temptation. Although mitigating the end-game effect is not a common practice in the PD literature, there are two prominent strategies to address the issue. Instead of explicitly defining the exact number of rounds to participants (finite round type) either this information is withheld, or the number of rounds is stochastically determined. The first can be achieved by simply not revealing to participants the total number of rounds of the game (Wedekind & Milinski, 1996; Melamed, Simpson & Harrell, 2017; Grujić & Lenaerts, 2020) while the second by pre-defining a specific probability for the game to be terminated after each round (Shafir & Tversky, 1992; Wedekind & Milinski, 1996; Kiyonari, Tanida & Yamagishi, 2000; Hehman, Flake & Freeman, 2018). Keeping the termination of the game vague, could help prevent the common human practice of defecting in the final rounds and thus avoid the ‘Nash equilibrium’ (Colman, Pulford & Krockow, 2018).

In the literature, the range of the total number of rounds used in experiments is vast, spanning from 2 to 500 with a median of 20 and an average value of around 45. The most commonly selected numbers of rounds which were used in more than half of the experiments, range between 10 and 30. In order to avoid end game effects, in 28 experiments, researchers have either chosen not to reveal the total number of rounds to participants or to assign, in each round, a specific probability for the game to be terminated (stochastic determination of the end of the game).

#### Instructions, narrative and options

The task’s instructions, narrative and options refer to the way and the context under which the PD game is presented to participants and are provided to participants before the beginning of the task or throughout the paradigm. The options that are available for the players and the representation of the payoffs matrix, the social context of the experiment’s implementation, the definition of the game’s purpose, the opponent type perception and knowledge of outcome’s inter-dependency (Martin et al., 2013) are aspects of the task that can influence the player’s decisions.

The typical PD narrative (criminal’s story), implements ‘cooperation’, ‘defect’ or ‘betray’ as available choices. This, however, is not the case for the majority of PD studies where letters (*i.e*., A/B, X/Y), numbers and other combinations are commonly used as analogues of cooperation and defection. These options are neutral enough to prevent priming competitive behaviour and also spare participants from the social moral weight of ‘defection’ or ‘betrayal’ which seem to promote cooperative decisions (Deutchman & Sullivan, 2018; Capraro et al., 2019), implement social bias (Capraro & Cococcioni, 2015) and are too diverse to represent common social interactions. Moreover, extra options (*i.e*., punishment, reward, withdrawal, 1–7) may be available to participants, either explicitly, as by payoff matrix definition, or more dynamically (*i.e*., ‘decide how many units to give to the other party’) depending on the PD design.

Researchers, when first introducing participants to the PD game, in addition to simply explaining the rational of the game and the matrix payoffs, which is the common practice, could use different imaginary scenarios (Table 1) in accordance with methods specific to the PD design. These scenarios usually lack the social bias of the original narrative but maintain their social context which contributes to the task’s understanding. It has been shown that socially-framed tasks are easier to grasp (Cosmides, 1989; Alekseev, Charness & Gneezy, 2017), and an in-depth comprehension of the task’s design and rules is crucial for the reliability of research. There is, however, the possibility of unexpected framing effects. For example, unpredictable associations between the imaginary scenarios presented in the PD experiment and past experiences of the players as well as intuitive or familiar preferences (Capraro, Jordan & Rand, 2014; Capraro & Cococcioni, 2015; Capraro et al., 2019). Additionally, Capraro & Rand (2018) and Capraro et al. (2019) investigating the role of morality as an influence in PD choices have shown that pro-social people tend to choose the option that is presented as being morally right in the given situation.

**Table 1:** This table summarized the different PD scenarios that are used in the literature.

Scenario	Description
Investment story	Participants are investors who have to choose between two investment projects, project X and Y.
Waitress tip story	Players must decide individually whether to contribute to a tip for the waiter.
Water shortage story	In a region suffering from drought, water is distributed by authorities in equal shares for each citizen. Players represent two different citizens who in order to obtain more supplies could pretend to represent the other citizen too. This succeeds only if one of the players complies else, if they try to cheat as well, both parties are punished by the authorities.
Boss-colleagues story	Players are colleagues who collaborated for a work project. Their boss is not satisfied with the quality and calls them individually into their office for explanations. There, the players have the choice to remain silent or blame each other.
Students’ copy story	Players take the role of students who copied at a test. Their professor notices that the tests are identical and convenes separately with each one telling them that if one of the two confesses (defection) the player will pass the test else they will have to wait for a number of sessions before taking it again.
Product offer story	Players take the role of owners of competing shops. They have to decide what price to assign to individual products. Either standard or sale (defection) price.
Sharing secrets story	Players, individually hide a secret in one of several boxes and have the opportunity to mutually plan their strategy for the trial. They have to decide, in private with the interviewer, whether or not to reveal (defection) to the interviewer the location of the secret. Even in the latter scenario, it is possible for the secret to be revealed by lack, as the interviewer can make one guess.
Arms race	Players play the role of a country leader deciding for their country’s resources. They can choose between military (missiles) or economic (factories) development. Houston et al. (2000) attached the dilemma to a 7 × 7 reward matrix so that missile building is awarded the most points if the other country built mostly factories but awarded no points if both parties built only missiles. In each round, players have to decide how many missiles, from zero to six, to invest with the rest to be invested in factories.
Doors-keys or Chests-keys (Brown & Rachlin, 1999)	The apparatus consists of two doors (or chests) of color X and two of color Y. Each one opens to a room with a reward and one key of color X or Y, both known to players. Opening a door, player A gets the reward, discards his current key and passes the revealed key to player B in order to make his door choice. The round finishes after both players get their reward with player A having the key which was inside the room opened by player B in the previous round. The above setting indicates a sequential PD. Each player defines the possible rewards of the other. The color of keys and the rewards are put in such a way that they form a classical PD reward matrix.
Give doubled (or tripled)	Each player, in each round is handed an amount of money and has to decide how much to give to the other. The amount given is doubled (or tripled). The formed matrix is dynamic and depends on decisions combination. Reward for player A equals: *n* − *n*A + 2 * *n*B (or *n* − *n*A + 3 * *n*B). with *n* the amount handled to each player at the beginning and *n*A, *n*B (*n* ≥ *n*A, *n* ≥ *n*B). The amounts given by players A and B respectively. If the available options are to give all or none, then the formed matrix is: T:3 * *n*, R:3 * *n*, P:1 * *n*, S:0 (or T:4 * *n*, R:3 * *n*, P:1 * *n*, S:0) where *n* is the amount handed to each player.
Leave doubled	Each player is handed with an amount each round and has to decide whether to leave or take other’s. The amount left is doubled for the opponent. The logic and rewards are the same as ‘give doubled’.
Give doubled—keep tripled	Each player is handed with an amount each round and has to decide how much to give to the other. The amount given is doubled for each one and the amount kept is tripled individually. The formed matrix is dynamic and depends on decisions combination. Reward for player A equals: 3 * (*n* − *n*A) + 2 * (*n*A + *n*B). with *n* the amount handed to each player at the beginning and *n*A, *n*B (*n* ≥ *n*A, *n* ≥ *n*B). the amounts given by players A and B respectively. If available options is to give all or none then the formed matrix is T:5 * *n*, R:4 * *n*, T:3 * *n*, S:2 * *n*.
Balls in baskets	Simple representation of ‘Give double—keep triple’. Putting a ball in basket A gives two points to all while putting it in basket B three to oneself.
Trays and beans	Apparatus consists of two similar, but independent, parallel et al., tilting trays with differently colored sides. Each player has control over one tray and has to decide between pulling his tray and gaining a reward that is stored in his side or pushing it thus giving their opponent the reward stored in the opposite side.
Joint invest	Each player is hande an amount for each round and has to decide how much to contribute, all or part of the amount, to an investment with the other player. The gathered amount is multiplied by a factor and distributed evenly between them. The formed matrix from decision combination is T:*n* + f * *n*/2, R:f * *n*, P:(*n* − *n*X) + f * *n*X/2, S:f * *n*/2 where *n*, *n*X(*n* > *n*X). Are the available investing options of the amount handed. In asymmetric PD, the factor (f) is different for each player leading to different reward matrices for each player.
Split or steal	Players mutually gather or are handed an amount. They have to decide whether to split it in half or steal the total. If they both steal, they get nothing. The formed matrix from decisions combination is T:2 * *n*, R: *n*, P:0, S:0 where 2 * *n* is the total amount.

**Note:**

A brief summary of the key elements of each PD scenario is offered in this table. The way each possible scenario could influence the outcome of the PD task is presented in the main text.

PD instructions commonly state that the purpose of the experiment is to achieve the maximum amount of points alternatively, they set the monetary reward as a function of the participants’ performance. This is done in order for the players to be motivated, the experiment to be an accurate representative of both real life and the human nature, and also to avoid the ‘always defect’ strategy which is the optimum for just winning but would not yield the maximum monetary rewards. Finally, to establish the adequate understanding of the task’s design and rules, most researchers require from participants to answer comprehension questions prior to PD trials.

Experimenters mainly prefer socially neutral (letters) or positive (cooperate *vs*. no-cooperate) vocabulary in their narrative as only 47 experiments use terms such as ‘defection’, ‘betray’, ‘punish’, ‘steal’ or ‘tell-on’ as options. A total of 19 experiments have implemented more than two options and 35 experiments utilised a different game design from the typical straightforward PD, like joint-invest, chest-keys etc.

#### Strategy

Strategy refers to the decision making process that unfolds during the PD game. The implementation of a strategy is necessary when the subject competes against an agent whose answers are controlled by the experimenter (algorithms). In ‘one-shot’ PD (see ‘number of rounds’ section), strategy implementation is limited to one decision since there is only one round. There are no priors of interaction and no planning involved as no future interaction will occur. This, however, is not the case for multiple ‘one-shot’ PDs which are played with the same opponent.

Strategy implementation mainly concerns the ‘iterated’ PD and by far the most common strategy is ‘tit-for-tat’ (Axelrod, 1984) and its variations. Other frequently used strategies include ‘always-cooperate’, ‘always-defect’ and ‘random’ (for a more detailed description see Table 2). ‘Tft’ starts with cooperation and then the strategy entails replicating the opponent’s previous move. In the famous Axelord’s competitions, this strategy has proven to be most successful while also promoting cooperation (Kuhlman & Marshello, 1975; Axelrod & Dion, 1988). ‘Tft’ has however, two main weaknesses, it cannot reverse an established mutual defection, meaning that any mistake or environmental noise would be of great influence during the course of the game and secondly, it cannot maximally exploit an unconditional cooperator. In order to eliminate the first weakness, two main subtypes of ‘tft’ have been proposed. The first subtype is called ‘forgiving-tft’ or ‘generous-tft’ (Nowak & Sigmund, 1992) and it involves a percentage of cooperation after the opponent’s defection. The second subtype is called ‘tit-for-two (or more)-tats’ (Boyd & Lorberbaum, 1987) where defection is chosen following continuous defections by the opponent.

**Table 2:** This table summarises all the available PD strategies used in the literature.

Strategy name	Description
Always Cooperate	Cooperates in each round.
Always Defect	Defects in each round.
Random	50% of cooperation or defection.
Tit for tat	Starts with cooperation and then the design replicate the opponents’ previous move.
*N*% Forgiving or Generous tit for tat	‘Tft’ with *N* percentage of cooperating after opponent’s defection.
Tit for *N* tats	‘Tft’ with cooperating until *N* number of consecutive opponent’s defections is reached.
Pavlov or Win-stay-Lose-swift	Switch choice whenever receives S or P payoffs.
Suspicious tit for tat	Like ‘tft’ but as ‘wsls’ does not swift on T payoff.
Rilling et al. (2002)	Cooperation in first round, defection in last three, else, decision based on last two players’ choices.
Rilling et al. (2004b)	50% possibility of reciprocate cooperation and always reciprocate defection.
Rilling et al. (2012)	For player A: 90% reciprocates defection and 67% reciprocates cooperation. For player B, ‘tft’ with 33% of cooperation after mutual defection, 10% of cooperation after unilateral (own) defection and always defection after two consecutive rounds of unilateral (own) defection.
McClure et al. (2007) (based on Rilling et al., 2002)	Cooperation in the first round, defection in the last two, 50% possibility of defection after four consecutive mutual cooperation rounds. Else, the decision is based on the player’s last two choices.
Zero Determinant (ZD) strategies (Press & Dyson, 2012)	Each decision is determined by the probabilities of cooperation based on the four possible choice combinations of the previous round.

**Note:**

A brief description of the key elements of each strategy is presented in this table. The impact that each strategy could have on the PD outcome is presented in the ’Strategy’ section of our review.

Another strategy which is underrepresented in the literature (Martin et al., 2013), is ‘win-stay-lose-shift’ (wsls) or ‘pavlov’ (Nowak & Sigmund, 1993) where the players switch their choice whenever they receive a bad payoff, S or P. ‘Wsls’ can be exploited by unconditional defectors but overcomes the ‘tft’ weaknesses. In order to promote cooperation, the reward matrix (see following section) in this strategy must fulfill the condition 2 * R > T + P where R stands for reward, T for temptation and P for punishment. Otherwise the player has no motive to cooperate (C) since continuous defection (D) (DD-DC loop) would gather at least the same amount of reward as cooperation (CC-CC or CD-CD).

Other interesting strategies mentioned in the literature are Zero Determinant (ZD) (Press & Dyson, 2012; Pansini, Shi & Wang, 2016), ‘pavlov-tft’ (Juvina et al., 2012) and ‘Raise the stakes’ (Roberts & Sherratt, 1998). ‘Pavlov-tft’ is considered to better fit human behaviour compared to its consisting strategies while ‘Raise the stakes’ can be applied to more dynamic invest-like PD designs. These designs, include ‘Give doubled—keep tripled’ and escalates the players’ investment when partners match or better their last move (for details on the different PD designs refer to Table 1). Other more straightforward, commonly implemented strategies include predefined choices (Papageorgiou et al., 2013), defecting in a number of rounds (Bitsch et al., 2018a) or decisions based on probabilities (*i.e*., 40% defection) (Haruno & Kawato, 2009). When PD is used for fMRI experiments, most strategies are based on Rilling et al. (2004b) work on collected human data and Rilling’s (Rilling et al., 2004a, 2004b, 2012) and McClure’s (McClure et al., 2007) strategies are the most frequently used, see Table 2.

As expected, the lion’s share in the medical literature (40 experiments) belongs to ‘tft’ based strategies while other ‘famous’ strategies where non-human participants are involved, like ‘wsls’ appear to be underrepresented (1 experiment). A total of 58 experiments involve non-typical and mixed strategies.

Table 2 summarizes the strategies used in literature.

#### Reward matrix and payoffs

The reward matrix and payoffs determine the gains and loses that different PD choices could bring to the players. The reward matrix could be considered the most important part of the PD as it determines the motive as well as the pros and cons of the agent’s decisions since different strategic choices are reflected upon the expected reward. There is no point in cooperating or defecting if there is no profit from it, or no punishment for doing otherwise. As a result, the amount of payoffs for each possible outcome and the degree of difference between these payoffs strongly influence the course of the game.

In a PD game, the reward matrix typically consists of four values, ‘T’ (temptation) is the reward of someone’s defection over cooperation, ‘R’ (reward) is the reward of mutual cooperation, ‘P’ (punishment) the payoff of mutual defection and ‘S’ (sucker) the payoff of cooperation over defection. In order for a reward matrix to accurately represent PD’s dilemma, the following condition must, at least, be met, T > R > P > S (Axelrod & Hamilton, 1981). The above is not sufficient for the ‘iterated’ version of the game, where 2 * R > T + S is another mandatory condition which gives mutual cooperation a greater reward value thus preventing individuals from alternating between cooperation and defection strategies (Axelrod & Hamilton, 1981). Moreover, as stated in the ‘strategy’ section, for the ‘wsls’ strategy to be meaningful, the 2 * R > T + P condition must also be met. Although the above conditions are standard for a typical PD game (Axelrod & Hamilton, 1981) a lot of papers deviate from these mainly by implementing the P < S condition, in order to promote cooperation (Bitsch et al., 2018b; Acosta, Straube & Kircher, 2019). The P < S condition, however, transforms such paradigms into a snowdrift or hawk-dove game which deviate from the typical PD task since mutual defection has the minimum payoff.

But how do the different combinations of those four matrix values, influence participants’ decisions? In order to answer that question, one factor that needs to be considered is the cooperation index ratio ((R − P )/(T − S)) used by Rapoport & Chammah (1965) and Rapoport (1967). Greater ratio values indicate that defection is less tempting and cooperation less risky, thus defining a more cooperation-friendly environment. In addition to the cooperation index ratio, Sherman (1967) proposes two other ratios derived from the values of the reward matrix parameters. These ratios represent attractiveness of cooperation ((R − P )/(P − S)) and attractiveness of defecting from a cooperative solution ((T − R)/(R − P )). A higher value for the first ratio represents relatively higher gain from cooperation while lower values for the second ratio relatively low gain from defecting, resulting, in both situations, in greater cooperation levels. The index of riskiness ((R − S)/((R − S) + (T − P ))), as proposed by Au et al. (2011) is a measure of the risk of cooperation, with values ranging from 0 to 1. Finally, Baas et al. (2019) and Huang et al. (2019) propose that (T − R) and (P − S) values could be representative of greed (gain the maximum from exploiting opponents cooperativeness) and threat (fear of being exploited) respectively, linking them both (mainly greed in older adults) to non-cooperative behavior.

Matrix payoffs are not just numbers, they represent units of reward. These rewards could either be real or hypothetical and whether that would influence the participants’ decisions remains unclear. PD experiments that tried to test this (Locey, Jones & Rachlin, 2011) produced ambivalent results and in general, the literature of decision making research examining hypothetical over real rewards has produced inconclusive results (Hertwig & Ortmann, 2001; Madden et al., 2003; Mieth et al., 2021). It is generally believed however, that real monetary rewards offer greater incentives compared to equivalent in value but hypothetical rewards (Hertwig & Ortmann, 2001; Bruner, Goodnow & Austin, 2017). However, how these would alter decisions in a PD setting, remains unknown. Nonetheless, Furlong and Offer suggest that what influences cooperation, is not the real value of payoffs but their numeric representation, meaning for example, that a reward of 300c would induce greater cooperational behaviour compared to that of 3$ (Furlong & Opfer, 2009).

In practice, in the literature, the most commonly encountered matrix values include quadruples T:3 R:2 P:1 S:0, T:5 R:3 P:1 S:0 and their multiples (*i.e*., T:30 R:20 P:10 S:0, T:15 R:9 P:3 S:0) as well as adding or subtracting a value from each of these payoff numbers (*i.e*., T:10 R:5 P:0 S:–5) (Supplemental Material).

Many experiments in the literature use negative values for P and S. Locey & Rachlin (2011) have shown that actual losses as punishment, lead to higher cooperation levels than gain reductions a finding that is consistent with previous literature (Gray & Tallman, 1987). Additionally, positive payoffs compared to losses (*i.e*., negative values for T, R, P and S), also favour cooperation (Sun et al., 2016; Deutchman & Sullivan, 2018).

Another aspect of the matrix that can influence the task’s outcome is the way it is presented (Evans & Crumbaugh, 1966) or in other words the context in which the conflict situation is presented to the participants. This could be either a grid representation of outcomes of every decision combination, *i.e*., T:3 R:2 P:1 S:0, or a non-grid representation, *i.e*., ‘either take 1 or give opponent 2’ which results to the same aforementioned grid. In Evans & Crumbaugh (1966), the non-grid representation which appeared to be better understood from participants, favoured cooperation.

Playing an ‘iterated’ PD of many rounds or multiple ‘one-shot’ games all with an unchanged reward matrix, can lead to boredom, loss of focus or strategic decisions that are made without adequate consideration. This is an important issue especially in psychophysiological experiments. Our literature search, revealed two ways to circumvent those issues and keep the participants interested in the task. Both ways involve the use of a dynamic matrix that entails changing payoffs in every round either by keeping the T − R difference the same (Emonds et al., 2013; Lambert et al., 2017) or by slightly altering the T, R and P values keeping however, the mean value of each parameter constant (Zhang et al., 2019).

Another reason for different amounts of reward associated with each round, is that the available amount might not be fixed for each round. This could be the case in more dynamic PD designs where the player decides how many units to give to the opponent (*i.e*., ‘Give doubled’, ‘Joint invest’) or in a network or multiplayer PD experiment where the final payoff of a round depends on the decision of other parties. Finally, in some cases, the payoffs can change as a result of decisions that occur between rounds such as the decision to punish the opponent after each round. (Fehl et al., 2012; Bone et al., 2015, 2016; Mieth et al., 2021). Payoffs can also change as the result of environmental variability, for example, a change in the reward values (Taheri, Rotshtein & Beierholm, 2018) or random decision switch (Rodebaugh et al., 2011, 2013; Grujić et al., 2012) can simulate uncertainty, which facilitates forgiveness and cooperation.

Based on payoff asymmetries, PD is distinguished in ‘symmetric’ and ‘asymmetric’. Most experiments implement a symmetric PD, there are however, a few experiments with asymmetric PD games (Gerbasi & Prentice, 2013; Aksoy & Weesie, 2014; Antonioni et al., 2018) where the reward asymmetry is used as a means of implementing inequality and hierarchy like in most social interactions.

Some PD literature uses matrices with more than four outcomes or in other words with a bigger payoff grid than 2 × 2. Wang et al. (2018) include for example, a reward option which plays the role of a more exploitable and beneficial for the opponent choice that is not associated with a greater payoff compared to cooperation. Pansini, Shi & Wang (2016) add the punishment option which is similar to cooperation in respect to one’s benefit but is most costly for the opponent. Finally, Insko et al. (2005) use the withdrawal option which results to the same payoff for both sides, irrespective of the opponents’ decision. Inserting one extra option as in the examples above leads to 9 (3 × 3 grid) possible rewards. Others (Houston et al., 2000; Knez & Camerer, 2000; Bone et al., 2016) use five or seven possible choices resulting to 25 or 49 possible payoffs. The rational behind these multichoice matrices is to create a reward gradient that could allow different cooperation and defection levels to be expressed without deviating too much from the typical PD game since taking into account only the first (defect) and last (cooperation) option of these multichoice matrices, still results in a valid typical PD.

## Conclusions

The PD game is one of the most popular concepts in medical literature as demonstrated by the vast number of related research. This review article focuses on the technical characteristics of the PD task and more specifically on the settings that are implemented in medical studies involving human subjects and how these could influence the task’s outcome.

The use of the PD in medical literature commonly evolves around the study of social decision making including cooperation (Testori, Hoyle & Eisenbarth, 2019), conflict (McClure et al., 2007) and moral judgement (Li, Zhu & Gummerum, 2014) as well as group dynamics and how these are affected in clinical populations (Testori, Hoyle & Eisenbarth, 2019) and/or after pharmacological intervention (Lane & Gowin, 2009; Rilling et al., 2012; Gabay et al., 2018). The PD game has also been used for the study of decision making and social characteristics in almost all psychiatric disorders, including psychotic and mood disorders, substance abuse and anxiety disorders (for relevant studies see McClure et al. (2007), Sorgi & Wout (2016), Bitsch et al. (2018b), Viola et al. (2019)). The majority of these studies however, focus on Autism Spectrum Disorder and personality traits where atypical social behaviour is a core characteristic (for studies using the PD on these disorders see Sheldon, Sheldon & Osbaldiston (2000), Halevy, Bornstein & Sagiv (2008), Locey, Safin & Rachlin (2012), Wang, Suri & Watts (2012), Li, Zhu & Gummerum (2014); De Dreu, Dussel & Velden, 2015, Weisel & Böhm (2015)).

Despite its widespread use in medical research, the PD game is not a diagnostic tool and no standardised form of the task exists. On the contrary, the parameters and settings of the task which comprise the paradigm’s technical characteristics and could significantly influence its outcome, vary depending on the focus of the research studies as well as the research population that is under investigation. On one hand, this “flexibility” in the PD settings allows investigators to adjust the paradigm in order to best address their research questions. On the other hand, however, it leads to a great heterogeneity that limits the reproducibility of the task’s outcomes and makes the experimental design of a research study involving the PD game a very arduous task.

In our review, we have focused on the typical one-*vs*.-one type of PD, as does the majority of the literature (207 out of 228 experiments) and identified, the seven parameters that need to be considered when setting up a PD experiment. For each parameter, we discuss the different settings that were used in the literature and how each one could influence the outcome of the experiment.

The vast majority of the medical research experiments utilise the PD task to examine cooperation between individuals with fewer studies looking at interaction between groups. More complicated designs of the opponent parties allow the examination of more complex aspects of human behaviour including altruism and social network dynamics.

Given the flexibility and reliability of having a computational experimental model, it is not surprising that the majority of the studies have used computers to conduct interactions and consequently, experimenters always have to consider whether or not to explicitly inform or even manipulate the subjects into believing they are competing against a real human opponent. Although when studying human behaviour, the influence of this factor remains controversial, most studies include such a manipulation in their experiments. However, when real human interactions are chosen, especially in a vis-a-vis setting, the role of the players’ emotions should also be taken into account since it has been shown that along with the players’ reputation and past behaviour they could greatly influence cooperation during the PD (de Melo & Terada, 2020).

As far as the interaction flow parameter is concerned, simultaneous PD which is the standard form and more accurately encompasses the nature of the dilemma is the most common type of PD. In the simultaneous PD the players are equals in terms of the game, and they just need to respond to the opponent’s move. Such a design is thus closer to the original PD design. In the sequential PD, however, the role of the players (player A or player B) defines their hierarchy and who would be controlling the game as in each round the player who responded last (Player B), has the advantage of knowing their opponent’s decision. This type of PD has been shown to promote cooperation. Cooperation is also favoured with ‘iterated’ PD which in its turn can be used to study more refined aspects of cooperation including the influence of hierarchy and reputation in decision making. In the case of an ‘iterated’ PD that consists of multiple, continuous ‘one-shot’ games the way in which the partners are matched is crucial. If partner matching is adopted, there is a high probability or even the certainty (fixed matching) of encountering the same opponent in a future match (Bó, 2005; Duffy & Ochs, 2009). In random matching however, this possibility is practically zero. Partner matching has a bold effect in the expected cooperation levels of ‘one shot’ PD (Rand & Nowak, 2013).

The total number of rounds is important only in ‘iterated’ PD since in order for strategies and cooperation to be established a sufficient number of rounds is required. Additionally, fewer rounds are associated with a higher risk of unsuccessful negotiation and loss and both factors should be considered when designing an ‘iterated’ PD experiment. Additionally, knowledge of the total number of rounds (finite *vs*. infinite round number) can alter someone’s strategy especially during the final rounds where the chance of retaliating decreases, this is referred to as the end game effect and is well documented (Lave, 1965; Selten & Stoecker, 1986; Normann & Wallace, 2011). To avoid end game effects, the final round of the game could be stochastically determined however, the efficiency of this approach in mitigating end-game effects is controversial. Dal Bo et al. have shown that the degree of cooperation between participants increases with increased continuation probability (Dal Bó & Fréchette, 2019), while Norman and Wallace concluded that any level of continuation probability does not significantly increase cooperation (Normann & Wallace, 2011).

In the literature, the instructions given to PD players are not well documented and, in the majority of the studies, they could only be inferred by looking at the description of the reward matrix. However, the instructions of the PD experiment are important since they determine the focus of the task and could influence the players’ behaviour. The context of the PD instructions, as stated in the relevant section, usually is deterring for defecting behaviours while at the same time, the traditional narrative of the PD instructions entails a high moral and social context. Such context is more comprehensive to participants and could be necessary depending on the experiment’s goal, for example when focus is given on measuring cooperation or studying other socially derived factors (Krach et al., 2008; Deutchman & Sullivan, 2018; Eimontaite et al., 2019).

Strategies only concern the ‘iterated’ version of PD. Algorithms are used to imitate human behaviour and to create a controlled environment for promoting and examining certain behaviours (*i.e*., cooperation) as well as for intra-individual correlations. The most common strategy appears to be ‘tft’, with the majority of research following non-typical and mixed strategies. The use of mixed and non-typical strategies not only contributes to more unwanted variability in research but also creates an extra difficulty concerning the categorization of these strategies, an issue that is not at all discussed in the literature. When classifying the studies of this review based on their strategy implementation, we were faced with the challenge of defining the margin over which a typical strategy fails to be representative of its family. For example, is a ‘tft’ strategy with standard defection in 10% of rounds still a ‘tft’ strategy? Keeping in mind that ‘suspicious-tft’ strategy, where there is a defection in the first round, is still considered a ‘tft’ strategy we have decided to set this cut off to two rounds.

The true essence of a PD game lays on its reward matrix which has to comply with certain rules (T > R > P > S, 2 * R > T + S). Most experiments use static matrices and positive values (T:3 R:2 P:1 S:0 rather that T:0 R:–1 P:–2 S:–3) with the most frequently chosen combinations these of T:3 R:2 P:1 S:0 and T:5 R:3 P:1 S:0 (Supplemental Material). It should be noted that oftentimes the literature associates the term PD with experiments such as snowdrift where the reward matrix does not adhere to the typical rules of a PD experiment, despite that, we have decided to include those experiments in our review. A lot of matrices use as rewards multiples of T:3 R:2 P:1 S:0 (and others) however, as mentioned in the relevant section, they have differential effects on the game, compared to the original matrices. Many PD experiments involved the use of multiple matrices (38 studies), that may even differ from round to round, or matrices with more than four outcomes (*i.e*., multiplayer PD). Beyond allowing the study of rewards and their effects, multiple matrices also serve motivational purposes or were a part of the game design chosen by the researchers, like in the case of asymmetrical PD.

Computers, as already mentioned, are the main medium, between the players and the researchers in a PD experiment. Computer applications implement all the parameters discussed in this review, along with the user interface, the time specifics (*i.e*., delays) and the interaction, delivering the final environment of each PD. ‘Ztree’, ‘EMOTICOM’, ‘E-Prime’, ‘MATLAB’, ‘AmazonTurk’ and ‘FlexibleIPD’ are some of the frameworks used for delivering PD tasks. In addition to the fact that there is no shared application between experiments, most of researchers do not include their application specifics. Given its crucial role, such information (*i.e*., algorithm for strategies simulation, images of the user interface, application’s source code) should at least be included in the Supplemental Material.

The seven parameters that we have identified and explained in our review could significantly influence the outcome of the PD task. The different settings that these parameters can take, make it very difficult to conclude to a unique set of parameters that would be optimal for PD medical research. Additionally, when PD research is published a lot of the crucial information around the task’s settings is not properly described making the replication of the PD findings and a consensus around the PD settings even more difficult to achieve. A shared common tool used for PD experiments in medical research would increase methodologies’ credibility, mitigate cross-validation discrepancies and relieve researchers from the difficulties of developing and testing yet another computer application.

## Supplemental Information

10.7717/peerj.12829/supp-1 Supplemental Information 1 PRISMA checklist.

10.7717/peerj.12829/supp-2 Supplemental Information 2 Summary of all the studies selected for this review. The 228 publications that were reviewed for our article are classified based on the choices that the experimenters have made for each of the seven parameters described in our review.

## List of abbreviations

PD: Prisoner’s Dilemma
T: Temptation Payoff
S: Sucker’s Payoff
P: Punishment Payoff
R: Reward Payoff
IPD-MD: Intergroup Prisoner’s Dilemma-Maximum Difference
tft: tit-for-tat
wsls: win-stay-lose-shift
ZD: Zero Determinant

## Additional Information and Declarations

### Data Availability

The following information was supplied regarding data availability: This is a literature review article. All the publications and data included in this review are presented in Table 1, Table 2 and the Supplementary Material.
