# Supplementary material for: Factors of influence in prisoner’s dilemma task: a review of medical literature
Source: PeerJ. 2022 Jan 28;10:e12829. doi: 10.7717/peerj.12829 (PMC8802712; doi:10.7717/peerj.12829)
Supplement: Supplemental Information 2 — The 228 publications that were reviewed for our article are classified based on the choices that the experimenters have made for each of the seven parameters described in our review. [file peerj-10-12829-s002.pdf]

reference No.	parties' composition				opponent perceived type				interaction flow type				instructions		opponents	rounds	strategy						reward matrix	comments
	group	multiplayer	individual		algorithm,predefined			real	simultaneous		sequential		narration/rules	options			tft[1]	wsls[2]	ac[3]	ad[4]	random /half[5]	other		[1]Tft: tit-for-tat [2]wsls: win-stay-lose-swift [3]ac: Always Coopereate [4]ad: Always Defetct [5]random: 50% of cooperation or defection, half: cooperate or defect in half of the rounds
			neighbours (links)	individual	non-human/pc		human		one-shot	iterated	one-shot	iterated												
					unknown, told non-human/pc	told human																		
Kumar et al., 2021				x			x		x				matrix	transfer/keep	4	1							6/4/2/0	
Zhang et al., 2020				x		x				x				red/black	1	60	x						10/7/0/0/, 10/3/0/0	
Cervantes Constantino et al., 2021				x			x			x			matrix	coop/not coop	1	200						x[1]	3/2/1/0	[1]c in round 1, reciprocation with 80%possibility, change of decision after 3 cosequtives same mutual decisions
Maeda and Hashimoto, 2020								x	x				Give doubled	give 3-5	1	1							depends on the amount given by each player	
Horita, 2020				x				x		x			Give doubled	give/retain	1	42							60/40/20/0	
Behrens et al., 2020				x				x		x			matrix	A,B,C,D,E,F	1	60							6x6 matrix	
Hobbs et al., 2020				x	x					x			matrix	coop/def	2	26	x[1]						75/50/25/0	[1]one of two opponents defected in the first round
de Melo and Terada, 2020				x		x				x			Investment story	project Green/Blue	1	20						x[1]	7/5/3/2	[1]extortionate/generous ZD strategy (Press and Dyson [ref.no 228])
Rostovtseva et al., 2020				x				x	x					coop/def	4	1								
Grujić and Lenaerts, 2020			x(4)					x		x			matrix	blue/yellow		50(unknown)							6/5/1/0	
Prétôt and McAuliffe, 2020				x				x		x			Tray and beans	blue/green or red/yellow	1	6							4/3/1/0	
Mieth et al., 2021				x	x						pA		Joint invest (factor:4/3)	coop/no coop	40	1							20/10/0/-10,10/5/0/-5	
Samu et al., 2020				x				x	x				matrix	L/D	20	1							2500/1500/500/0	
Prétôt et al., 2020				x				x	x				Tray and beans	blue/green or red/yellow	7	1							4/3/1/0	
Koch et al., 2020				x		x			x				Give doubled	give 0-1 (step 0.1)	30	1							depends on the amount given by each player	
Neto et al., 2020				x	x	x						pA,pB		coop/def	2	30						x[1]	3/2/1/0	[1]Rilling 2012 [ref.no 155]
Lin et al., 2020				x		x			x				matrix	A/B	30	1							15/10/5/0	
Cigarini et al., 2020				x				x	x				matrix (sharing resources)	coop/def	2	1							15/10/5/0,3/2/1/0	
Gallotti and Grujić, 2019			x(8)					x		x			matrix	blue/yellow	fixed - reshuffled type(multiplayer), 1(vs individual)	random-unknown(47,60,58) (multiplayer),100(vs individual)							10/7/0/0	
V.Capraro, 2019				x				x	x				Give doubled	transfer/not transfer	1	1							30/20/10/0	
Chen et al., 2019		X(3)						x	x					Strategy A,B	12[1,2]	1							22/20/18/16/14/12 based on the combination of choices[3]	[1]random regroup after each round [2]opponents decisions are anonymously presented [3]possibility of punishment after each round for a player of own or other group
Huang et al., 2019				x		x			x					coop/no coop	32	1							1.4/1/0.6/0.2, 1.4/1/0.6/0.5, 1.1/1/0.6/0.2, 1.1/1/0.6/0.5	
Javor et al., 2019				x		x				x				coop/no coop	4	32(unknown)						x[1]	50/30/10/0	[1]Rilling 2002 [ref.no 200]
Yang and Yue, 2019				x	x			x	x	x				A/B	<=5,<=15[1]	1,<=25			x				12/8/3/1	[1] possible opponent change in each round based on the previous 5 choices
Heuer and Orland, 2019			x(10)					x	x				matrix	A/B		1							85/75/30/25	
Eimontaite et al., 2019				x						x			Prisoner's story	keep silent/tell on	9	12							6 matrices: 5*n/3*n/-1*n/-5*n	
Thompson et al., 2019				x		x				x				coop/no coop	3	20						x[1]	3/2/1/0	[1]McClure 2007 [ref.no 183]
Viola et al., 2019				x					x				Prisoner's story										3/2/1/0	
Zhang et al., 2019				x		x				x				coop/def	1	240							3/2/1/0 [1]	[1]changing matrix with stable mean reward values
Testori et al., 2019			x(<=2)					x		x				coop/nothing		50(unknown)							coop: costs of 10 per neighbor while neighbors gain 60 each	
Mienaltowski and Wichman, 2020				x		x				x			Doors-keys	select door	1	32	x					x[1]		[1]self-interest on 25% of rounds
Baas et al., 2019				x									matrix	C/NC		5							6/4/3/2, 6/4/3/0	
Rodriguez-Ruiz et al., 2019				x			x	x	x						1	1							160/90/30/10	
Chu et al., 2019				x				x					matrix	split/steal	1				x[1]				10/5/1/0	[1]d in round 1
Testori et al., 2019				x	x	x				x			Product offer story	standard price/sale price	1	30	tf2t[1]						40/30/20/10	[1]d in 2 last rounds
Schaper et al., 2019				x	x				x				Joint invest (factor:4/3)	coop/no coop	60	1							20/10/0/-10	
Belaus et al., 2018				x				x		x				A/B	1	1								
Savulich et al., 2018				x	x					x			Split or steal	split/steal	3		x,tf2t		x				2*n/n/0/0 2*n:points gathered	
Acosta et al., 2019				x		x				x				left/right	2	60						x[1]	20/20/0/10	[1]Rilling ,2004a [ref.no 195]
Galbiati et al., 2018				x				x	x	x			matrix	A/B	until 15min concluded	75% possibility for next round							60/40/35/12, 50/40/25/12	
Gabay et al., 2018				x		x				x				compete/coop	6	15					x	x[1,2]	120/90/60/30	[1]c in 13/15 rounds [2]c in 3/15 rounds
Zhang et al., 2018				x	x									red/black card	1		x						10/7/3/0	
Taheri et al., 2018				x	x			x		x				collaborate/betray	2	13-17						x[1]	0.5/0.3/0.1/0 [2]	[1]Tft with random choice at 20% of rounds [2]possible loss or gain of 0.2 after a round
Dorrough and Glöckner, 2018				x				x	x				Give doubled	transfer 0-200 (step 10)	1	6							30/20/10/0	
Bitsch et al., 2018a				x		x				x				C/D	3	6						x[1,2,3]	20/20/0/10	[1]c in 5 rounds [2]c in 3rounds [3]d in 5 rounds
Deutchman and Sullivan, 2018				x		x			x				Prisoner's story, matrix	coop/betray, A/B	1	1							20/10/15/0, 0/-10/-15/-20	
Wang et al., 2018				x				x		x			matrix	1/2/3	~20(until 75min)	~4(25% possibility next round)							3/1/0/-2 [1]	[1]reward option costs 2 for player and gains 3 for the opponent

Acedo-Carmona and Gomila, 2018				x			x			x			matrix	share/get	2	3	x		x				6/3/1/0	
Vives and FeldmanHall, 2018				x					x														5/4/1/0	
Arechar et al., 2018				x				x	x	x			matrix	A/B	many	7/8,1/8 next round							5/4/1/0	
Levine et al., 2018				x		x					pA,pB		Give doubled	transfer/not transfer	1	1							90/60/30/0	
Colman et al., 2018				x				x		x			matrix	J/K	1	300							5/3/1/0,5/4/1/0	
Bruno et al., 2018	x							x		x					1	11								
Antonioni et al., 2018				x				x		x			matrix	X/Y	1	10(unknown)							4*n/3*n/2*n/1*n [1]	[1]asymmetric PD, n based on hierarchy for each player
Bitsch et al., 2018b				x		x				x			matrix	coop/def	21	6						x[1,2,3]	20/20/0/10	[1]c in 5 rounds [2]c in 3rounds [3]d in 5 rounds
Li et al., 2018				x			x			x			matrix	triangle/circle shape [1]	1	10	x						4/3/2/1	[1]punishment option for the cooperator after round
Rilling et al., 2018				x	x	x						pA,pB		coop/def	4	30						x[1]	3/2/1/0	[1]Rilling 2012 [ref.no 155]
Hu et al., 2018				x				x[1]		x					1	120							6/5/1/0,7/4/1/-1	[1]half told opponent is the computer
Menshikov et al., 2017				x				x	x	x					3	18							10/5/1/0	
Kaartinen et al., 2017				x		x			x		pB		matrix	keep/give	2	1							3/2/1/0	
Bell et al., 2017				x	x						pA		Joint invest (factor:4/3)	coop/no coop	48	1							20/10/0/-10,10/5/0/-5	
Bland et al., 2017				x	x					x			Split or steal	split/steal	3	9	tf2t, stft		x				2*n/n/0/0, 2*n:points gathered	
Jahng et al., 2017				x				x(live)		x				coop/def	1	30(unknown)							10/5/3/0	
Barreda-Tarrazona et al., 2017				x				x	x	x			matrix	A/B	10(one-shot),2(iterated)	1(one-shot),10(iterated)							28/20/10/0,2.8/2/1/0	
Peshkovskaya et al., 2017				x				x	x	x				up/down,left/right	<=11(one-shot),1(iterated)	1(one-shot),15(iterated)							10/5/1/0	
Melamed et al., 2017				x				x	x	x			matrix	contribute/not contribute	>=3	12(unknown)							7/4/1/-1	
Chen et al., 2017				x	x	x						pA,pB		coop/def	2	30						x[1]	3/2/1/0	[1]Rilling 2012 [ref.no 155]
Lambert et al., 2017				x		x					pA			K/L	25	120(randomly distributed among opps)						x[1]	25 different matrices with standard T-R=1	[1]60% c after c, 100% d after d
Mieth et al., 2016a				x	x				x				Joint invest (factor:4/3)	coop/not coop [1]	20								20/10/0/-10	[1]punishment option for the cooperator after round
Pansini et al., 2016				x				x		x				coop/def/punish	many	75% next round							3/1/0/-2 [1]	[1]punishment option costs 1 for player and 6 for the opponent
Sorgi and Wout, 2016				x		x				x				coop/def	3	12						x[1,2,3]	10/6/2/0,5/3/1/0	[1]c in 25% of rounds [2]c in 50% of rounds [3]c in 75% of rounds
Rawolle et al., 2016				x		x				x				coop/no-coop	1	10				x[1]			65/60/20/15	[1]c in 2 rounds
Mieth et al., 2016b				x	x						pA		Joint invest (factor:4/3)	invest 15/30	20	1							15/10/5/0	
Sun et al., 2016				x		x			x					C/D	80	1							30/20/10/0,0/-10/-20/-30	
Lukinova and Myagkov, 2016				x		x								up/down	?	30trials						x[1]	6/4/2/1	[1]d in 85% of rounds and c in 15%
Matsumoto et al., 2016				x		x			x	x	pA,pB		Give doubled	amount to give	9	1							depends on the amount given by each player	
Yao and Yu, 2016				x		x				x				C/D	1	12						x[1]	8/4/-1/-5	[1]d in rounds 1,5 and 9
Antonioni et al., 2016			x(2,3,4)					x		x			matrix	A/B		30(unknown)							10/7/0/0	
Bone et al., 2016				x				x		x			matrix	interact time(0-5) [1]	2	50(unknown)							5x5 matrix	[1]punishment option after round with different cost per player type
Schneider-Hassloff et al., 2016				x	?				x					?	2	1							6/5/1/0	
Wang et al., 2016				x	x	x				x			matrix	C/D	2	60,500(unknown)						x[1]	5/3/1/0	[1]extortionate/generous ZD strategy (Press and Dyson [ref.no 228])
Bell et al., 2016				x		x					pA		Joint invest (factor:4/3)	invest 15/30	18	1							10/5/-5/-10	
Collins et al., 2016				x			x			x			matrix	A/B	1-2	50	x[1]					Pavlov-tft	4/1/-1/-4	[1]random choice in two rounds
Fermin et al., 2016				x		x					pA		Give doubled	provide/not provide (100/200/400)	15	1							3*n/2*n/n/0, n{100.200.400}	
Luo et al., 2016				x	x					x			Prisoner's story	coop/def	1	136					x		0/-3/-8/-10	
Etzel et al., 2016				x			x			x			matrix	C/D	2	3						x[1,2]	3/2/1/0, 4/2/0/-1, 5/3/-1/-3 [3]	[1]cooperating strategy [2]competing strategy [3]one matrix per round
Gradin et al., 2016				x		x				x				coop/no coop	1	76						x[1]	3/2/1/0	[1]McClure 2007 [ref.no 183]
Wildschut et al., 2015																								
Bell et al., 2015				x		x					pA	pA	Joint invest (factor:4/3)	invest 15/30	150	1,4							15/10/5/0	
Weisel and Böhm, 2015	x[1]							x																[1]Intergroup Prisoner's Dilemma-Maximizing Difference (IPD-MD)
Ciardo et al., 2015				x		x			x				Students' copy story	coop/compete	8	1							0/-1/-6/-7	
Soutschek et al., 2015				x	x					x				coop yes/no	4	12						x[1]	3/2/0/-1	[1]Tft with 20% possibility of changing decision
Ng and Au, 2015				x					x					A/B	28	1							28 matrices[1]	[1]different coop [ref.no 229] and risk [ref.no 230] index values
C. Feng et al., 2015				x	x	x						pA,pB		cop/def	2	30(pA),30(pB)						x[1]		[1]Rilling 2012 [ref.no 155]
Schneider-Hassloff et al., 2015				x		x						pA		left/right	2	10						x[1]	20/20/10/0	[1]Rilling 2004a [ref.no 195]
Capraro and Cococcioni, 2015				x		x			x				Give doubled	transfer (0-20)	1	1							depends on the amount given by each player	
Reimers and Diekhof, 2015				x		x			x				matrix	coop/def	40	1							60/40/20/0	
Chen et al., 2015				x	x	x						pA,pB	matrix	coop/def	2	30(pA),30(pB)						x[1]	3/2/1/0	[1]Rilling 2012 [ref.no 155]
Dreu et al., 2015	x[1]																							[1]IPD-MD
Chunliang Feng et al., 2015					x	x						pA,pB		coop/def	2	30(pA),30(pB)						x[1]	3/2/1/0	[1]Rilling 2012 [ref.no 155]
Dorrough et al., 2015				x				x	x				Give doubled	transfer (0-10)	20	1								
Safin et al., 2015				x	x					x				blue/green	2	40					x		10/9/2/1,4/3/2/1	

Pinel et al., 2015				x		x				x				A/B		10			x				4/3/1/0	
Saunders et al., 2015				x		x				x				coop/def	1	40(20x2)	x,stft					x[1]	70/40/20/0	
Soutschek et al., 2015				x	x					x				coop yes/no	1	98,140,195						x[1]	3/2/0/-1	[1]Tft with 20% possibility of changing decision
Bone et al., 2015				x				x		x			matrix	A/B/not participate [1]	2	50(unknown)							3/1/0/-2	[1]punishment option after round with different cost per player type
V.Capraro, 2014				x				x	x				Give [2,3,4,5,10]fold	transfer (0-10)	1	1							depends on the number the transferred amount is multiplied and the amount transferred by each player	
Edmiston et al., 2014				x		x				x				coop/compete	1	20						x[1]	3/2/1/0	[1]Rilling 2002 [ref.no 200]
Christensen et al., 2014								x		x			Sharing Secrets story	interviewer correct/incorrect	1								0/-45/-30/-60	
Capraro and Marcelletti, 2014				x		x			x				Give doubled	hand over/do not	1	1							0.6/0.4/0.2/0	
Feng et al., 2014				x	x	x						pA,pB		coop/def	2	30(pA),30(pB)							3/2/1/0	[1] Rilling 2012 [ref.no 155]
Johnstone et al., 2015				x	x					x					2	15							6/5/1/0	
Pulcu et al., 2014				x	x					x			Water shortage story		1	31	tf2t						8/5/1/0	
Bednarik et al., 2014				x				x		x				orange/blue	3+	30(unknown)							0.4/0.25/0/-0.1	
Yamagishi et al., 2014				x				x	x		pA,pB		Give doubled		9	1							3*n/2*n/n/0	
Clark et al., 2014				x				x		x				coop/def	1	10	x						5/3/1/0	
Mengel, 2014				x					x				matrix	A/B	10	1							450/400/200/100,450/400/120/100	
Carolyn Henriette Declerck et al., 2013				x					x		pB			L/S	2	1							12/8/4/1	
Cárdenas et al., 2014				x				x		x			Balls in baskets	private/public basket	1	10							5/4/3/2	
Li et al., 2014				x				x		x			matrix	circle/triangular	1	10	x						4/3/2/1	
Aksoy and Weesie, 2014				x							pA,pB		Joint invest	invest/not invest	4(pA),4(pB)	1							8 different reward matrices[1]	[1]Asymmetric PD
Kjell and Thompson, 2013				x		x				x				A/B	1	10	x						3/2/1/0	
Sakaiya et al., 2013				x	x	x				x				coop/def	4	20-23(unknown)	x				x		50/30/10/0	
Emonds et al., 2013				x		x			x					K/L	25	1					x[1]		25 different matrices	[1]Rilling 2004b [ref.no 227]
Rilling et al., 2014				x	x	x						pA,pB		coop/def	2	30(pA),30(pB)						x[1]	3/2/1/0	[1]Rilling 2012 [ref.no 155]
Kovács-Bálint et al., 2012				x	x				x				Prisoner's story	coop/no coop	1	1								
Acedo and Gomila, 2013				x				x		x				C/D	1	3							6/3/1/0	
Eimontaite et al., 2013				x			x			x			Prisoner's story	tell on/keep silent	1	3							50000/30000/-10000/-5000	
Safin et al., 2013				x				x		x			matrix	green/blue circles	1	40							4/3/2/1, 10/9/2/1	
Papageorgiou et al., 2013				x	x					x				coop/def	1	90(unknown)						x[1]	5/3/1/0	[1]Tft with predefined C in rounds 5,10,20,30,40,50,60,70,80,90
Gerbasi and Prentice, 2013				x		x				x				A/B	1	10							p:400/200/100/0 opp:200/100/50/0[1]	[1]Asymmetric PD
Clark et al., 2013				x			x			x					1	10	x						5/3/1/0	
Carolyn H. Declerck et al., 2013				x			x		x		pB			L/S	2	1							12/8/4/1	
Tabak et al., 2013				x	x					x				C/D	1	20-40						x[1]	3/2/1/0	[1]50%ftft for the 1-12 rounds, D for 7 rounds after 12 consecutive rounds of cooperation
Gervais et al., 2013				x				x	x				Give doubled	give/keep 3	2	1							9/6/3/0	
Storey and Workman, 2013				x				x		x				coop/def	1	5								
Locey et al., 2012		x(5,10,20)		x				x	x				matrix	X/Y	1	1							10/9/2/1, 4/3/2/1, rewards based on how many players chose Y (Y:nY*100, X:nY*100+300)	
Rodebaugh et al., 2013				x	x					x			Give doubled - keep tripled	keep/share (1-10) [2]	2	40	x					x[1]	depends on the amount given by each player	[1]Raise the Stakes [ref.no 161] [2]change of player's decision (adding or subtracting 3) in 10% of rounds
Yamagishi et al., 2012				x				x																
Tayama et al., 2012				x			x			x				Card 1/2	1	<=100[1]						x[2]	5/4/-4/-5	[1]End of game determined by difference in combination of DD and CC (Punishment-score), [2]Tft with increasing cooperation after eight D of player
Yang et al., 2013				x	x				x							1							5/3/1/0	
Fehl et al., 2012				x				x	x				Joint invest (factor:1.6)	contribute/not 1E [1]	5	1							0.8/0.6/0/-0.2	[1]punishment option after round
Grujić et al., 2012		x(<=5)						x		x			matrix	A/B [1]									rewards based on how many players chose A (A:(nA-1)*7, B:nA*10)	[1]random change of player's decision
Wang et al., 2012			x(<24)					x		x			matrix	A/B		12							7/4/1/-1	
Locey and Rachlin, 2012					x					x				blue/red	1	100	x						30/25/15/10	
Martin et al., 2013	x			x	x			x					matrix	action A/B	1	200	x	x	x	x			10/1/-1/-10	
Gracia-Lazaro et al., 2012			x(2-16)					x		x			matrix	brown/green		51,59							10/7/0/0	
Locey and Rachlin, 2011				x	x					x			matrix	X/Y	1	100	x						5/4/2/1,2/1/-1/-2	
Ellett et al., 2013				x	x	x			x					X/Y	1	1(unknown)							120/90/60/30	
Pfeiffer et al., 2012				x				x		x				A/B	1,many	29(unknown),4							30/20/0/-10	
Locey et al., 2011					x								Chests-keys	select chest	0	40	x						4/3/2/1,6/5/2/1	
Ewoldsen et al., 2012				x				x		x			Give doubled	give dimes 0-4	1	10							depends on the amount given by each player	
McClure et al., 2013				x		x			x					strategy A/B	4	1							6/4/2/0	
Glöckner and Hilbig, 2012				x				x		x				1/2	many	20							300/200/100/50,300/varied/varied/50	
Emonds et al., 2012				x		x			x					invest/not	28	1							3/2/1/0,6/5/1/0,2/1/-1/-2,8/5/2/0	

Balliet et al., 2011				x			x			x			matrix	option A/B	1	2(unknown)				x			100/60/40/0	
Rilling et al., 2012				x	x	x						pA,pB		coop/def	2	30(pA),30(pB)						x[1]	3/2/1/0	[1]Rilling 2012 [ref.no 155]
Volstorf et al., 2011				x	x								Boss-colleagues story	coop/refuse	20	10(unknown)	x			x			5/3/1/0	
McClure-Tone et al., 2011				x		x				x				coop/no-coop	4	20						x[1]	3/2/1/0	[1]McClure 2007 [ref.no 183]
Dijk et al., 2011				x	x					x				coop/def	1	2					x		5/3/1/0	
Tabak et al., 2012				x		x				x				?	1	34	50% ftft		x	x			3/2/1/0	
Fallani et al., 2010				x				x						coop/def	1	200							3/2/1/0	
Rodebaugh et al., 2011				x	x					x			Give doubled - keep tripled	keep/share (1-10)	1	40						x[1]	depends on the amount given by each player	[1]FickleFriend: Raise the stakes with D in the 20% of last rounds [2]change of player's decision (adding or subtracting 3) in 10% of rounds. required prediction.
Grujić et al., 2010			x(8)					x		x				blue/yellow		47,58,60							10/7/0/0	
Astolfi et al., 2010				x				x	x					coop/def	1	1								
Suzuki et al., 2010				x	x	x				x				coop/def	6	18						x[1]	25/20/0/-5	[1]C decision based on previous opponents decisions such as P(c)=0.1+0.8*(nC/(nC+nD))
Declerck et al., 2010				x		x			x					L/S	1	1							12/8/4/1	
Stevens et al., 2011				x	x					x			Waitress tip story	coop/no coop	5,10,15	5,10,15							5/3/1/0	
Lane and Gowin, 2009				x	x					x				A/C	4	100					x		0.5/0.25/-0.25/0	
Fischer, 2009				x				x	x					&/#	1	1							20/14/2/0,20/10/5/0	
Kircher et al., 2009				x	x	x				x				?	3						x		20/10/0/0	
Haruno and Kawato, 2009				x	x	x				x				coop/def	2	18	x					x[1]	50/30/-20/-30	[1]70% probability of C
Krach et al., 2009				x	x	x				x				coop/def	2	90					x		20/10/0/0	
Lount et al., 2008				x		x				x			Tutorial of cooperation [1]	X/Y	1	30			x[2]				30/24/12/6	[1]Murnighan, 1991, pp. 13-27 [2]All C except for D in two rounds
Furlong and Opfer, 2009				x				x		x				coop/def	1	80	x						5/3/1/0$,500/300/100/0c,5/3/1/0c,500/300/100/0$,5/3/1/0,1005/1003/1001/1000,105/103/101/100,0.05/0.03/0.01/0,0.005/0.003/0.001/0	
Lönnqvist et al., 2009				x				x	x				Give doubled, Leave doubled	transfer to other 1-10, transfer to me 1-10	2	1							depends on the amount given by each player	
Chater et al., 2008				x		x			x					1/2	19	1							21/20/2/1, 0/-31/-34/-35, 36/5/2/1, 11/10/1/0, 0/-5/-6/-11	
Krach et al., 2008				x	x			x					Prisoner's story	coop/def	4	10					x		20/20/10/0	
Mokros et al., 2008				x	x					x			Water shortage story	coop/def	1	40	tf2t						8/5/1/0	
Halevy et al., 2008	x[1]							x						keep/A/B										[1]IPD-MD
Wolf et al., 2008	x			x				x		x			matrix	X/Y	1	5							10/90/60/30	
Rilling et al., 2004a				x		x				x				coop/def	2	20	33% ftft[1]					x[2]	3/2/1/0	[1]D in first round, [2]Rilling 2002 [ref.no 200]
Kümmerli et al., 2007				x				x		x				C/D	1	12							400/300/0/-100	
Hopthrow et al., 2007	x			x				x						J/P	1	4	x						60/50/15/10	
McClure et al., 2007				x		x				x				coop/no coop	4	20						x[1]	3/2/1/0	[1]McClure 2007 [ref.no 183]
Takemura and Yuki, 2007	x			x				x		x				X/Y	1	10							11 matrices: 4*n/3*n/2*n/n	
Rilling et al., 2007				x	x					x				coop/def	2	20	33% ftft						3/2/1/0	
Yi et al., 2007				x	x					x				coop/def	1	60	x						25/20/10/5	
Johnson et al., 2004				x				x		x				X/Y	1	6							10/8/3/0,0/-2/-7/-10	
Eek and Gärling, 2006				x					x				matrix	A/B	1	1							400/200/100/50	
West et al., 2006				x				x		x				coop/def	2	7(unknown)							5/3/2/1	
Wood et al., 2006				x		x						pB		coop/def	2	20	x,sftf						30/20/10/0	
Yi et al., 2005				x	x					x				coop/def	2	60	x				x		25/20/10/5	
Wong and Hong, 2005				x				x	x					Strategy A/B	5	1								
Insko et al., 2005	x				x				x					X/Y/Z	1	1							360/270/180/90 [1]	[1]withdrawal option with ensured reward of 225 for both players
Yi and Rachlin, 2004		x(5)				x				x			matrix	X/Y			x				x		rewards based on how many players chose Y (X:nY*3+7, Y: nY*3)	
Rilling et al., 2004b				x	x	x					pA			coop/def	28	1							6/5/1/0	
Rotenberg et al., 2004				x				x						green/red light	<=62								15/10/?/5	
Liberman et al., 2004				x				x		x				C/D		5,7							80/40/0/-20,8/6/-6/-8	
Singer et al., 2004				x		x						pA	Give tripled		>22	4								
Ketelaar and Au, 2003				x		x				x				A/B	8	10	x					x[1,2]	5/3/1/0	[1]C in first five rounds and tft afterwards [2]D in first five rounds and tft afterwards
Rilling et al., 2002				x	x	x		x		x				coop/def	3	20-23(known, unknown)	stft					x[1,2]	3/2/1/0	[1]C in first five rounds and tft afterwards [2]Rilling 2002 [ref.no 200]
Wildschut et al., 2002	x					x			x					X/Y		1							500/500/200/200,500/490/110/100,500/400/200/100	
Baker and Rachlin, 2002				x								pB		left/right	1	100						x[1,2]	6/5/2/1	[1]Tft with changing probability of C based on the cooperational behavior of the player [2]Wsls with changing probability of C based on the cooperational behavior of the player
Kiyonari et al., 2000				x			x		x		pA,pB			K/P	3	1				x			188/1200/600/0	
Sheldon et al., 2000		x(2,3,4)						x	x					coop/get ahead		1							rewards based on how many players chose C (C:nC*2, G:nC*2+5)	
Houston et al., 2000				x		x				x			Arms race	0-6 missiles	1	5						x[1,2,3]	7x7matrix	[1]competitive strategy [2]deescalating strategy [3]noncompetitive strategy
Knez and Camerer, 2000				x				x		x				1/2/3/4/5/6/7	1	5							3x3,7x7 matrix	required prediction

Brown and Rachlin, 1999				x				x					Doors-keys	select door	2	20								
Milinski and Wedekind, 1998				x			x			x				C/D	2	~20(unknown)			x			x[1]	4/3/1/0	[1]tft until 5th round and D afterwards
Wedekind and Milinski, 1996				x			x							C/D	2	~20(unknown)			x			x[1]	4/3/1/0	[1]tft until 5th round and D afterwards
Kiesler et al., 1996				x	x		x						Investment story	project Green/Blue	4	6							7/5/4/3	
Shafir and Tversky, 1992				x		x			x				matrix	coop/compete	6	1							?	
Lindskold and Han, 1988				x		x				x				choice1 /2	1	20						x[1]	5/4/-4/-5	[1] Random until 10th round and tf2t afterwards
Smith and Brehm, 1981				x			x			x				cooperative/competitive	1	20							10/2/-2/-10	
Podd et al., 1970				x		x				x				?	1	45						x[1]		[1]15 rounds C, 15 rounds Random and 15 rounds D
Swingle, 1968				x		x				x				1/2	2	5,10,20,40,60						x[1,2]	4/3/1/0	[1]5% possibility of C [2]95% possibility of C
Tedeschi et al., 1968				x				x		x				?	1	100					x		10/1/-1/-10	
Guyer, 1968				x				x		x				X/Y	1	300							different matrix according to algorithm with input player's decisions.	
Sermat, 1967				x		x				x				red/black	1	70						x[1]	10/8/3/1	[1]D until 50th round and C afterwards
Crumbaugh and Evans, 1967				x				x		x			matrix	A/B,give me/him	1	50	x					x	4/3/1/0	
Arnstein and Feigenbaum, 1967				x			x			x				A/B	1	24	x						7/5/1/-2	
Swingle and Coady, 1967				x		x				x				1/2	1	100			x[1]	x[1]	x[1]	x[2]	4/3/1/0	[1]All-defection until 50th round and change to one of the other to the end [2]25% possibility of C
Rapoport and Mowshowitz, 1966				x	x		x	x		x			matrix	C/D	1	1h						x[1]	10/1/5/-10	[1]based on Markov chain strategy
Evans and Crumbaugh, 1966				x				x		x			matrix	A/B,give me 1/him 3	1	50	x						4/3/1/0	
Rapoport and Mowshowitz, 1966				x				x		x			matrix	C/D	1	300							7 matrices	
Komorita, 1965				x				x		x				red/black	1	80							5/3/0/-1	
Lave, 1965				x			x							strategy 1/2	1	100,50,25,15			x	x		x[1,2]	6 matrices	[1]D in almost all rounds [2]D unless player decides C five times

Table 1 includes that 228 studies that are part of this review. For each study, the choice that experimenters made for each of the seven parameters is indicated.

References

1. Acedo, C., Gomila, A., 2013. Trust and cooperation: a new experimental approach. Annals of the New York Academy of Sciences 1299, 77–83. https://doi.org/10.1111/nyas.12142
2. Acedo-Carmona, C., Gomila, A., 2018. Personal trust extends cooperation beyond trustees: A Mexican study. International Journal of Psychology 54, 687–704. https://doi.org/10.1002/ijop.12500
3. Acosta, H., Straube, B., Kircher, T., 2019. Schizotypy and mentalizing: An fMRI study. Neuropsychologia 124, 299–310. https://doi.org/10.1016/j.neuropsychologia.2018.11.012
4. Aksoy, O., Weesie, J., 2014. Hierarchical Bayesian analysis of outcome- and process-based social preferences and beliefs in Dictator Games and sequential Prisoner's Dilemmas. Social Science Research 45, 98–116. https://doi.org/10.1016/j.ssresearch.2013.12.014
5. Antonioni, A., Pereda, M., Cronin, K.A., Tomassini, M., Sánchez, A., 2018. Collaborative hierarchy maintains cooperation in asymmetric games. Scientific Reports 8. https://doi.org/10.1038/s41598-018-23681-z
6. Antonioni, A., Sánchez, A., Tomassini, M., 2016. Cooperation Survives and Cheating Pays in a Dynamic Network Structure with Unreliable Reputation. Scientific Reports 6. https://doi.org/10.1038/srep27160
7. Arechar, A., Kouchaki, M., Rand, D., 2018. Examining Spillovers between Long and Short Repeated Prisoner's Dilemma Games Played in the Laboratory. Games 9, 5. https://doi.org/10.3390/g9010005
8. Arnstein, F., Feigenbaum, K.D., 1967. Relationship of Three Motives to Choice in the Prisoner's Dilemma. Psychological Reports 20, 751–755. https://doi.org/10.2466/pr0.1967.20.3.751
9. Astolfi, L., Cincotti, F., Mattia, D., Fallani, F.D.V., Salinari, S., Vecchiato, G., Toppi, J., Wilke, C., Doud, A., Yuan, H., He, B., Babiloni, F., 2010. Simultaneous estimation of cortical activity during social interactions by using EEG hyperscannings, in: 2010 Annual International Conference of the IEEE Engineering in Medicine and Biology. IEEE. https://doi.org/10.1109/iembs.2010.5626555
10. Au, W.T., Lu, S., Leung, H., Yam, P., Fung, J.M.Y., 2011. Risk and Prisoner's Dilemma: A Reinterpretation of Coombs' Re-parameterization. Journal of Behavioral Decision Making 25, 476–490. https://doi.org/10.1002/bdm.743
11. Baas, M., Roskes, M., Koch, S., Cheng, Y., De Dreu, C.K.W., 2019. Why Social Threat Motivates Malevolent Creativity. Pers Soc Psychol Bull 45, 1590–1602. https://doi.org/10.1177/0146167219838551
12. Baker, F., Rachlin, H., 2002. Teaching and learning in a probabilistic prisoner's dilemma. Behavioural Processes 57, 211–226. https://doi.org/10.1016/s0376-6357(02)00015-3
13. Balliet, D., Li, N.P., Joireman, J., 2011. Relating trait self-control and forgiveness within prosocials and proselfs: Compensatory versus synergistic models. Journal of Personality and Social Psychology 101, 1090–1105. https://doi.org/10.1037/a0024967
14. Barreda-Tarrazona, I., Jaramillo-Gutiérrez, A., Pavan, M., Sabater-Grande, G., 2017. Individual Characteristics vs. Experience: An Experimental Study on Cooperation in Prisoner's Dilemma. Frontiers in Psychology 8. https://doi.org/10.3389/fpsyg.2017.00596
15. Bednarik, P., Fehl, K., Semmann, D., 2014. Costs for switching partners reduce network dynamics but not cooperative behaviour. Proceedings of the Royal Society B: Biological Sciences 281, 20141661. https://doi.org/10.1098/rspb.2014.1661
16. Behrens, F., Snijdewint, J.A., Moulder, R.G., Prochazkova, E., Sjak-Shie, E.E., Boker, S.M., Kret, M.E., 2020. Physiological synchrony is associated with cooperative success in real-life interactions. Sci Rep 10, 19609. https://doi.org/10.1038/s41598-020-76539-8
17. Belaus, A., Reyna, C., Freidin, E., 2018. Testing the effect of cooperative/competitive priming on the Prisoner's Dilemma. A replication study. PLOS ONE 13, e0209263. https://doi.org/10.1371/journal.pone.0209263
18. Bell, R., Koranyi, N., Buchner, A., Rothermund, K., 2016. The implicit cognition of reciprocal exchange: automatic retrieval of positive and negative experiences with partners in a prisoner's dilemma game. Cognition and Emotion 31, 657–670. https://doi.org/10.1080/02699931.2016.1147423
19. Bell, R., Mieth, L., Buchner, A., 2017. Separating conditional and unconditional cooperation in a sequential Prisoner's Dilemma game. PLOS ONE 12, e0187952. https://doi.org/10.1371/journal.pone.0187952
20. Bell, R., Sasse, J., Möller, M., Czernochowski, D., Mayr, S., Buchner, A., 2015. Event-related potentials in response to cheating and cooperation in a social dilemma game. Psychophysiology 53, 216–228. https://doi.org/10.1111/psyp.12561
21. Bitsch, F., Berger, P., Nagels, A., Falkenberg, I., Straube, B., 2018a. Impaired Right Temporoparietal Junction–Hippocampus Connectivity in Schizophrenia and Its Relevance for Generating Representations of Other Minds. Schizophrenia Bulletin 45, 934–945. https://doi.org/10.1093/schbul/sby132
22. Bitsch, F., Berger, P., Nagels, A., Falkenberg, I., Straube, B., 2018b. The role of the right temporo-parietal junction in social decision-making. Human Brain Mapping 39, 3072–3085. https://doi.org/10.1002/hbm.24061
23. Bland, A.R., Roiser, J.P., Mehta, M.A., Schei, T., Sahakian, B.J., Robbins, T.W., Elliott, R., 2017. Cooperative Behavior in the Ultimatum Game and Prisoner's Dilemma Depends on Players' Contributions. Frontiers in Psychology 8. https://doi.org/10.3389/fpsyg.2017.01017
24. Bone, J.E., Wallace, B., Bshary, R., Raihani, N.J., 2016. Power Asymmetries and Punishment in a Prisoner's Dilemma with Variable Cooperative Investment. PLOS ONE 11, e0155773. https://doi.org/10.1371/journal.pone.0155773
25. Bone, J.E., Wallace, B., Bshary, R., Raihani, N.J., 2015. The Effect of Power Asymmetries on Cooperation and Punishment in a Prisoner's Dilemma Game. PLOS ONE 10, e0117183. https://doi.org/10.1371/journal.pone.0117183
26. Brown, J., Rachlin, H., 1999. Self-control and social cooperation. Behavioural Processes 47, 65–72. https://doi.org/10.1016/s0376-6357(99)00054-6
27. Bruno, A., Dell'Aversana, G., Guidetti, G., 2018. Developing Organizational Competences for Conflict Management: The Use of the Prisoner's Dilemma in Higher Education. Frontiers in Psychology 9. https://doi.org/10.3389/fpsyg.2018.00376
28. Capraro, V., Cococcioni, G., 2015. Social setting, intuition and experience in laboratory experiments interact to shape cooperative decision-making. Proceedings of the Royal Society B: Biological Sciences 282, 20150237. https://doi.org/10.1098/rspb.2015.0237
29. Capraro, V., Jagfeld, G., Klein, R., Mul, M., de Pol, I. van, 2019. Increasing altruistic and cooperative behaviour with simple moral nudges. Sci Rep 9, 11880. https://doi.org/10.1038/s41598-019-48094-4
30. Capraro, V., Jordan, J.J., Rand, D.G., 2014. Heuristics guide the implementation of social preferences in one-shot Prisoner's Dilemma experiments. Sci Rep 4, 6790. https://doi.org/10.1038/srep06790
31. Capraro, V., Marcelletti, A., 2014. Do good actions inspire good actions in others? Scientific Reports 4. https://doi.org/10.1038/srep07470
32. Cárdenas, J.-C., Dreber, A., Essen, E. von, Ranehill, E., 2014. Gender and Cooperation in Children: Experiments in Colombia and Sweden. PLoS ONE 9, e90923. https://doi.org/10.1371/journal.pone.0090923
33. Cervantes Constantino, F., Garat, S., Nicolaisen-Sobesky, E., Paz, V., Martínez-Montes, E., Kessel, D., Cabana, Á., Gradin, V.B., 2021. Neural processing of iterated prisoner's dilemma outcomes indicates next-round choice and speed to reciprocate cooperation. Social Neuroscience 1–18.
34. Chater, N., Vlaev, I., Grinberg, M., 2008. A new consequence of Simpson's paradox: Stable cooperation in one-shot prisoner's dilemma from populations of individualistic learners. Journal of Experimental Psychology: General 137, 403–421. https://doi.org/10.1037/0096-3445.137.3.403
35. Chen, S., Shi, J., Yang, X., Ye, H., Luo, J., 2019. Modulating Activity in the Dorsolateral Prefrontal Cortex Changes Punishment in the 3-Player Prisoner's Dilemma: A Transcranial Direct Current Stimulation Study. Front. Neurosci. 13. https://doi.org/10.3389/fnins.2019.01160
36. Chen, X., Gautam, P., Haroon, E., Rilling, J.K., 2017. Within vs. between-subject effects of intranasal oxytocin on the neural response to cooperative and non-cooperative social interactions. Psychoneuroendocrinology 78, 22–30. https://doi.org/10.1016/j.psyneuen.2017.01.006
37. Chen, X., Hackett, P.D., DeMarco, A.C., Feng, C., Stair, S., Haroon, E., Ditzen, B., Pagnoni, G., Rilling, J.K., 2015. Effects of oxytocin and vasopressin on the neural response to unreciprocated cooperation within brain regions involved in stress and anxiety in men and women. Brain Imaging and Behavior 10, 581–593. https://doi.org/10.1007/s11682-015-9411-7
38. Christensen, J.C., Shiyanov, P.A., Estepp, J.R., Schlager, J.J., 2014. Lack of Association between Human Plasma Oxytocin and Interpersonal Trust in a Prisoner's Dilemma Paradigm. PLoS ONE 9, e116172. https://doi.org/10.1371/journal.pone.0116172
39. Chu, V.C., Lucas, G.M., Lei, S., Mozgai, S., Khooshabeh, P., Gratch, J., 2019. Emotion Regulation in the Prisoner's Dilemma: Effects of Reappraisal on Behavioral Measures and Cardiovascular Measures of Challenge and Threat. Front. Hum. Neurosci. 13. https://doi.org/10.3389/fnhum.2019.00050
40. Ciardo, F., Ricciardelli, P., Lugli, L., Rubichi, S., Iani, C., 2015. Eyes keep watch over you! Competition enhances joint attention in females. Acta Psychologica 160, 170–177. https://doi.org/10.1016/j.actpsy.2015.07.013
41. Cigarini, A., Vicens, J., Perelló, J., 2020. Gender-based pairings influence cooperative expectations and behaviours. Sci Rep 10, 1041. https://doi.org/10.1038/s41598-020-57749-6
42. Clark, B.C., Thorne, C.B., Hendricks, P.S., Sharp, C., Clark, S.K., Cropsey, K.L., 2014. Individuals in the criminal justice system show differences in cooperative behaviour: Implications from cooperative games. Criminal Behaviour and Mental Health 25, 169–180. https://doi.org/10.1002/cbm.1920
43. Clark, C.B., Thorne, C.B., Hardy, S., Cropsey, K.L., 2013. Cooperation and depressive symptoms. Journal of Affective Disorders 150, 1184–1187. https://doi.org/10.1016/j.jad.2013.05.011
44. Collins, M.G., Juvina, I., Gluck, K.A., 2016. Cognitive Model of Trust Dynamics Predicts Human Behavior within and between Two Games of Strategic Interaction with Computerized Confederate Agents. Frontiers in Psychology 7. https://doi.org/10.3389/fpsyg.2016.00049
45. Colman, A.M., Pulford, B.D., Krockow, E.M., 2018. Persistent cooperation and gender differences in repeated Prisoner's Dilemma games: Some things never change. Acta Psychologica 187, 1–8. https://doi.org/10.1016/j.actpsy.2018.04.014
46. Crumbaugh, C.M., Evans, G.W., 1967. Presentation Format, Other-Person Strategies, and Cooperative Behavior in the Prisoner's Dilemma. Psychological Reports 20, 895–902. https://doi.org/10.2466/pr0.1967.20.3.895
47. de Melo, C.M., Terada, K., 2020. The interplay of emotion expressions and strategy in promoting cooperation in the iterated prisoner's dilemma. Sci Rep 10, 14959. https://doi.org/10.1038/s41598-020-71919-6
48. Declerck, Carolyn Henriette, Boone, C., Kiyonari, T., 2013. No Place to Hide: When Shame Causes Proselfs to Cooperate. The Journal of Social Psychology 154, 74–88. https://doi.org/10.1080/00224545.2013.855158

49. Declerck, Carolyn H., Boone, C., Kiyonari, T., 2013. The effect of oxytocin on cooperation in a prisoner's dilemma depends on the social context and a person's social value orientation. Social Cognitive and Affective Neuroscience 9, 802–809. https://doi.org/10.1093/scan/nst040
50. Declerck, C.H., Boone, C., Kiyonari, T., 2010. Oxytocin and cooperation under conditions of uncertainty: The modulating role of incentives and social information. Hormones and Behavior 57, 368–374. https://doi.org/10.1016/j.yhbeh.2010.01.006
51. Deutchman, P., Sullivan, J., 2018. The Dark Triad and framing effects predict selfish behavior in a one-shot Prisoner's Dilemma. PLOS ONE 13, e0203891. https://doi.org/10.1371/journal.pone.0203891
52. Dijk, C., Koenig, B., Ketelaar, T., Jong, P.J. de, 2011. Saved by the blush: Being trusted despite defecting. Emotion 11, 313–319. https://doi.org/10.1037/a0022774
53. Dorrough, A.R., Glöckner, A., 2018. A cross-national analysis of sex differences in prisoner's dilemma games. British Journal of Social Psychology 58, 225–240. https://doi.org/10.1111/bjso.12287
54. Dorrough, A.R., Glöckner, A., Hellmann, D.M., Ebert, I., 2015. The development of ingroup favoritism in repeated social dilemmas. Frontiers in Psychology 6. https://doi.org/10.3389/fpsyg.2015.00476
55. Dreu, C.K.W.D., Dussel, D.B., Velden, F.S.T., 2015. In intergroup conflict, self-sacrifice is stronger among pro-social individuals, and parochial altruism emerges especially among cognitively taxed individuals. Frontiers in Psychology 06. https://doi.org/10.3389/fpsyg.2015.00572
56. Edmiston, E.K., Merkle, K., Corbett, B.A., 2014. Neural and cortisol responses during play with human and computer partners in children with autism. Social Cognitive and Affective Neuroscience 10, 1074–1083. https://doi.org/10.1093/scan/nsu159
57. Eek, D., Gärling, T., 2006. Prosocials prefer equal outcomes to maximizing joint outcomes. British Journal of Social Psychology 45, 321–337. https://doi.org/10.1348/014466605x52290
58. Eimontaite, I., Nicolle, A., Schindler, I., Goel, V., 2013. The effect of partner-directed emotion in social exchange decision-making. Frontiers in Psychology 4. https://doi.org/10.3389/fpsyg.2013.00469
59. Eimontaite, I., Schindler, I., De Marco, M., Duzzi, D., Venneri, A., Goel, V., 2019. Left Amygdala and Putamen Activation Modulate Emotion Driven Decisions in the Iterated Prisoner's Dilemma Game. Front. Neurosci. 13. https://doi.org/10.3389/fnins.2019.00741
60. Ellett, L., Allen-Crooks, R., Stevens, A., Wildschut, T., Chadwick, P., 2013. A paradigm for the study of paranoia in the general population: The Prisoner's Dilemma Game. Cognition & Emotion 27, 53–62. https://doi.org/10.1080/02699931.2012.689757
61. Emonds, G., Declerck, C.H., Boone, C., Seurinck, R., Achten, R., 2013. Establishing cooperation in a mixed-motive social dilemma. An fMRI study investigating the role of social value orientation and dispositional trust. Social Neuroscience 9, 10–22. https://doi.org/10.1080/17470919.2013.858080
62. Emonds, G., Declerck, C.H., Boone, C., Vandervliet, E.J.M., Parizel, P.M., 2012. The cognitive demands on cooperation in social dilemmas: An fMRI study. Social Neuroscience 7, 494–509. https://doi.org/10.1080/17470919.2012.655426
63. Etzel, J.A., Valchev, N., Gazzola, V., Keysers, C., 2016. Is Brain Activity during Action Observation Modulated by the Perceived Fairness of the Actor? PLOS ONE 11, e0145350. https://doi.org/10.1371/journal.pone.0145350
64. Evans, G.W., Crumbaugh, C.M., 1966. Effects of prisoner's dilemma format on cooperative behavior. Journal of Personality and Social Psychology 3, 486–488. https://doi.org/10.1037/h0023035
65. Ewoldsen, D.R., Eno, C.A., Okdie, B.M., Velez, J.A., Guadagno, R.E., DeCoster, J., 2012. Effect of Playing Violent Video Games Cooperatively or Competitively on Subsequent Cooperative Behavior. Cyberpsychology, Behavior, and Social Networking 15, 277–280. https://doi.org/10.1089/cyber.2011.0308
66. Fallani, F.D.V., Nicosia, V., Sinatra, R., Astolfi, L., Cincotti, F., Mattia, D., Wilke, C., Doud, A., Latora, V., He, B., Babiloni, F., 2010. Defecting or Not Defecting: How to "Read" Human Behavior during Cooperative Games by EEG Measurements. PLoS ONE 5, e14187. https://doi.org/10.1371/journal.pone.0014187
67. Fehl, K., Sommerfeld, R.D., Semmann, D., Krambeck, H.-J., Milinski, M., 2012. I Dare You to Punish Me—Vendettas in Games of Cooperation. PLoS ONE 7, e45093. https://doi.org/10.1371/journal.pone.0045093
68. Feng, Chunliang, DeMarco, A.C., Haroon, E., Rilling, J.K., 2015. Neuroticism modulates the effects of intranasal vasopressin treatment on the neural response to positive and negative social interactions. Neuropsychologia 73, 108–115. https://doi.org/10.1016/j.neuropsychologia.2015.05.004
69. Feng, C., Hackett, P.D., DeMarco, A.C., Chen, X., Stair, S., Haroon, E., Ditzen, B., Pagnoni, G., Rilling, J.K., 2014. Oxytocin and vasopressin effects on the neural response to social cooperation are modulated by sex in humans. Brain Imaging and Behavior 9, 754–764. https://doi.org/10.1007/s11682-014-9333-9
70. Feng, C., Lori, A., Waldman, I.D., Binder, E.B., Haroon, E., Rilling, J.K., 2015. A common oxytocin receptor gene (OXTR) polymorphism modulates intranasal oxytocin effects on the neural response to social cooperation in humans. Genes, Brain and Behavior 14, 516–525. https://doi.org/10.1111/gbb.12234
71. Fermin, A.S.R., Sakagami, M., Kiyonari, T., Li, Y., Matsumoto, Y., Yamagishi, T., 2016. Representation of economic preferences in the structure and function of the amygdala and prefrontal cortex. Scientific Reports 6. https://doi.org/10.1038/srep20982
72. Fischer, I., 2009. Friend or foe: Subjective expected relative similarity as a determinant of cooperation. Journal of Experimental Psychology: General 138, 341–350. https://doi.org/10.1037/a0016073
73. Furlong, E.E., Opfer, J.E., 2009. Cognitive Constraints on How Economic Rewards Affect Cooperation. Psychological Science 20, 11–16. https://doi.org/10.1111/j.1467-9280.2008.02244.x
74. Gabay, A.S., Kempton, M.J., Gilleen, J., Mehta, M.A., 2018. MDMA Increases Cooperation and Recruitment of Social Brain Areas When Playing Trustworthy Players in an Iterated Prisoner's Dilemma. The Journal of Neuroscience 39, 307–320. https://doi.org/10.1523/jneurosci.1276-18.2018
75. Galbiati, R., Henry, E., Jacquemet, N., 2018. Dynamic effects of enforcement on cooperation. Proceedings of the National Academy of Sciences 115, 12425–12428. https://doi.org/10.1073/pnas.1813502115
76. Gallotti, R., Grujić, J., 2019. A quantitative description of the transition between intuitive altruism and rational deliberation in iterated Prisoner's Dilemma experiments. Sci Rep 9, 17046. https://doi.org/10.1038/s41598-019-52359-3
77. Gerbasi, M.E., Prentice, D.A., 2013. The Self- and Other-Interest Inventory. Journal of Personality and Social Psychology 105, 495–514. https://doi.org/10.1037/a0033483
78. Gervais, M.M., Kline, M., Ludmer, M., George, R., Manson, J.H., 2013. The strategy of psychopathy: primary psychopathic traits predict defection on low-value relationships. Proceedings of the Royal Society B: Biological Sciences 280, 20122773. https://doi.org/10.1098/rspb.2012.2773
79. Glöckner, A., Hilbig, B.E., 2012. Risk is relative: Risk aversion yields cooperation rather than defection in cooperation-friendly environments. Psychonomic Bulletin & Review 19, 546–553. https://doi.org/10.3758/s13423-012-0224-z
80. Gracia-Lazaro, C., Ferrer, A., Ruiz, G., Tarancon, A., Cuesta, J.A., Sanchez, A., Moreno, Y., 2012. Heterogeneous networks do not promote cooperation when humans play a Prisoner's Dilemma. Proceedings of the National Academy of Sciences 109, 12922–12926. https://doi.org/10.1073/pnas.1206681109
81. Gradin, V.B., Pérez, A., Macfarlane, J.A., Cavin, I., Waiter, G., Tone, E.B., Dritschel, B., Maiche, A., Steele, J.D., 2016. Neural correlates of social exchanges during the Prisoner's Dilemma game in depression. Psychological Medicine 46, 1289–1300. https://doi.org/10.1017/s0033291715002834
82. Grujić, J., Eke, B., Cabrales, A., Cuesta, J.A., Sánchez, A., 2012. Three is a crowd in iterated prisoner's dilemmas: experimental evidence on reciprocal behavior. Scientific Reports 2. https://doi.org/10.1038/srep00638
83. Grujić, J., Fosco, C., Araujo, L., Cuesta, J.A., Sánchez, A., 2010. Social Experiments in the Mesoscale: Humans Playing a Spatial Prisoner's Dilemma. PLoS ONE 5, e13749. https://doi.org/10.1371/journal.pone.0013749
84. Grujić, J., Lenaerts, T., n.d. Do people imitate when making decisions? Evidence from a spatial Prisoner's Dilemma experiment. Royal Society Open Science 7, 200618. https://doi.org/10.1098/rsos.200618
85. Guyer, M., 1968. Response-dependent parameter changes in the prisoner's dilemma game. Behavioral Science 13, 205–219. https://doi.org/10.1002/bs.3830130305
86. Halevy, N., Bornstein, G., Sagiv, L., 2008. "In-Group Love" and "Out-Group Hate" as Motives for Individual Participation in Intergroup Conflict. Psychological Science 19, 405–411. https://doi.org/10.1111/j.1467-9280.2008.02100.x
87. Haruno, M., Kawato, M., 2009. Activity in the Superior Temporal Sulcus Highlights Learning Competence in an Interaction Game. Journal of Neuroscience 29, 4542–4547. https://doi.org/10.1523/jneurosci.2707-08.2009
88. Heuer, L., Orland, A., 2019. Cooperation in the Prisoner's Dilemma: an experimental comparison between pure and mixed strategies. Royal Society Open Science 6, 182142. https://doi.org/10.1098/rsos.182142
89. Hobbs, C., Murphy, S.E., Wright, L., Carson, J., Assche, I.V., O'Brien, J., Oyesanya, M., Sui, J., Munafò, M.R., Kessler, D., Harmer, C.J., Button, K.S., 2020. Effect of acute citalopram on self-referential emotional processing and social cognition in healthy volunteers. BJPsych Open 6, e124. https://doi.org/10.1192/bjo.2020.107
90. Hopthrow, T., Abrams, D., Frings, D., Hulbert, L.G., 2007. Groupdrink: The effects of alcohol on intergroup competitiveness. Psychology of Addictive Behaviors 21, 272–276. https://doi.org/10.1037/0893-164x.21.2.272
91. Horita, Y., 2020. Greater effects of mutual cooperation and defection on subsequent cooperation in direct reciprocity games than generalized reciprocity games: Behavioral experiments and analysis using multilevel models. PLOS ONE 15, e0242607. https://doi.org/10.1371/journal.pone.0242607
92. Houston, J.M., Kinnie, J., Lupo, B., Terry, C., Ho, S.S., 2000. Competitiveness and Conflict Behavior in Simulation of a Social Dilemma. Psychological Reports 86, 1219–1225. https://doi.org/10.2466/pr0.2000.86.3c.1219
93. Hu, Y., Pan, Y., Shi, X., Cai, Q., Li, X., Cheng, X., 2018. Inter-brain synchrony and cooperation context in interactive decision making. Biological Psychology 133, 54–62. https://doi.org/10.1016/j.biopsycho.2017.12.005
94. Huang, Y., Chai, J., Feng, L., Yu, R., 2019. Older Adults Show Diminished Sensitivity to Potential Losses in Social Bargaining. The Journals of Gerontology: Series B. https://doi.org/10.1093/geronb/gbz113
95. Insko, C.A., Kirchner, J.L., Pinter, B., Efaw, J., Wildschut, T., 2005. Interindividual-Intergroup Discontinuity as a Function of Trust and Categorization: The Paradox of Expected Cooperation. Journal of Personality and Social Psychology 88, 365–385. https://doi.org/10.1037/0022-3514.88.2.365
96. Jahng, J., Kralik, J.D., Hwang, D.-U., Jeong, J., 2017. Neural dynamics of two players when using nonverbal cues to gauge intentions to cooperate during the Prisoner's Dilemma Game. NeuroImage 157, 263–274. https://doi.org/10.1016/j.neuroimage.2017.06.024
97. Javor, A., Ciumas, C., Ibarrola, D., Ryvlin, P., Rheims, S., 2019. Social cognition, behaviour and therapy adherence in frontal lobe epilepsy: a study combining neuroeconomic and neuropsychological methods. Royal Society Open Science 6, 180850. https://doi.org/10.1098/rsos.180850
98. Johnson, T., Myagkov, M., Orbell, J., 2004. Sociality as a defensive response to the threat of loss. Politics and the Life Sciences 23, 13–19. https://doi.org/10.2990/1471-5457(2004)23[13:saadrt]2.0.co;2
99. Johnstone, B., Cohen, D., Bryant, K.R., Glass, B., Christ, S.E., 2015. Functional and structural indices of empathy: Evidence for self-orientation as a neuropsychological foundation of empathy. Neuropsychology 29, 463–472. https://doi.org/10.1037/neu0000155
100. Kaartinen, M., Puura, K., Pispa, P., Helminen, M., Salmelin, R., Pelkonen, E., Juujärvi, P., Kessler, E.B., Skuse, D.H., 2017. Associations between cooperation, reactive aggression and social impairments among boys with autism spectrum disorder. Autism 23, 154–166. https://doi.org/10.1177/1362361317726417
101. Ketelaar, T., Au, W.T., 2003. The effects of feelings of guilt on the behaviour of uncooperative individuals in repeated social bargaining games: An affect-as-information interpretation of the role of emotion in social interaction. Cognition and Emotion 17, 429–453. https://doi.org/10.1080/02699930143000662
102. Kiesler, S., Sproull, L., Waters, K., 1996. A prisoner's dilemma experiment on cooperation with people and human-like computers. Journal of Personality and Social Psychology 70, 47–65. https://doi.org/10.1037/0022-3514.70.1.47
103. Kircher, T., Blümel, I., Marjoram, D., Lataster, T., Krabbendam, L., Weber, J., Os, J. van, Krach, S., 2009. Online mentalising investigated with functional MRI. Neuroscience Letters 454, 176–181. https://doi.org/10.1016/j.neulet.2009.03.026
104. Kiyonari, T., Tanida, S., Yamagishi, T., 2000. Social exchange and reciprocity: confusion or a heuristic? Evolution and Human Behavior 21, 411–427. https://doi.org/10.1016/s1090-5138(00)00055-6
105. Kjell, O.N.E., Thompson, S., 2013. Exploring the impact of positive and negative emotions on cooperative behaviour in a Prisoner's Dilemma Game. PeerJ 1, e231. https://doi.org/10.7717/peerj.231
106. Knez, M., Camerer, C., 2000. Increasing Cooperation in Prisoner's Dilemmas by Establishing a Precedent of Efficiency in Coordination Games. Organizational Behavior and Human Decision Processes 82, 194–216. https://doi.org/10.1006/obhd.2000.2882
107. Koch, A., Dorrough, A., Glöckner, A., Imhoff, R., 2020. The ABC of society: Perceived similarity in agency/socioeconomic success and conservative-progressive beliefs increases intergroup cooperation. Journal of experimental social psychology 90, 103996.
108. Komorita, S.S., 1965. Cooperative choice in a Prisoner's Dilemma game. Journal of Personality and Social Psychology 2, 741–745. https://doi.org/10.1037/h0022673
109. Kovács-Bálint, Z., Bereczkei, T., Hernádi, I., 2012. The telltale face: Possible mechanisms behind defector and cooperator recognition revealed by emotional facial expression metrics. British Journal of Psychology n/a-n/a. https://doi.org/10.1111/bjop.12007
110. Krach, S., Blümel, I., Marjoram, D., Lataster, T., Krabbendam, L., Weber, J., Os, J. van, Kircher, T., 2009. Are women better mindreaders? Sex differences in neural correlates of mentalizing detected with functional MRI. BMC Neuroscience 10. https://doi.org/10.1186/1471-2202-10-9
111. Krach, S., Hegel, F., Wrede, B., Sagerer, G., Binkofski, F., Kircher, T., 2008. Can Machines Think? Interaction and Perspective Taking with Robots Investigated via fMRI. PLoS ONE 3, e2597. https://doi.org/10.1371/journal.pone.0002597
112. Kumar, M.M., Tsoi, L., Lee, M.S., Cone, J., McAuliffe, K., 2021. Nationality dominates gender in decision-making in the Dictator and Prisoner's Dilemma Games. PLOS ONE 16, e0244568. https://doi.org/10.1371/journal.pone.0244568
113. Kümmerli, R., Colliard, C., Fiechter, N., Petitpierre, B., Russier, F., Keller, L., 2007. Human cooperation in social dilemmas: comparing the Snowdrift game with the Prisoner's Dilemma. Proceedings of the Royal Society B: Biological Sciences 274, 2965–2970. https://doi.org/10.1098/rspb.2007.0793
114. Lambert, B., Declerck, C.H., Emonds, G., Boone, C., 2017. Trust as commodity: social value orientation affects the neural substrates of learning to cooperate. Social Cognitive and Affective Neuroscience 12, 609–617. https://doi.org/10.1093/scan/nsw170
115. Lane, S.D., Gowin, J.L., 2009. GABAergic modulation of human social interaction in a prisoner's dilemma model by acute administration of alprazolam. Behavioural Pharmacology 20, 657–661. https://doi.org/10.1097/fbp.0b013e32832ec62b
116. Lave, L.B., 1965. Factors affecting co-operation in the prisoner's dilemma. Behavioral Science 10, 26–38. https://doi.org/10.1002/bs.3830100104
117. Levine, E.E., Barasch, A., Rand, D., Berman, J.Z., Small, D.A., 2018. Signaling emotion and reason in cooperation. Journal of Experimental Psychology: General 147, 702–719. https://doi.org/10.1037/xge0000399
118. Li, J., Zhu, L., Chen, Z., 2018. The association between punishment and cooperation in children with high-functioning autism. Journal of Experimental Child Psychology 171, 1–13. https://doi.org/10.1016/j.jecp.2018.01.015
119. Li, J., Zhu, L., Gummerum, M., 2014. The relationship between moral judgment and cooperation in children with high-functioning autism. Scientific Reports 4. https://doi.org/10.1038/srep04314
120. Liberman, V., Samuels, S.M., Ross, L., 2004. The Name of the Game: Predictive Power of Reputations versus Situational Labels in Determining Prisoner's Dilemma Game Moves. Personality and Social Psychology Bulletin 30, 1175–1185. https://doi.org/10.1177/0146167204264004
121. Lin, Y., Hu, P., Mai, Z., Jiang, T., Mo, L., Ma, N., 2020. Sleep Deprivation Impairs Cooperative Behavior Selectively: Evidence from Prisoner's and Chicken Dilemmas. Nature and science of sleep 12, 29.
122. Lindskold, S., Han, G., 1988. GRIT as a Foundation for Integrative Bargaining. Personality and Social Psychology Bulletin 14, 335–345. https://doi.org/10.1177/0146167288142011
123. Locey, M.L., Jones, B.A., Rachlin, H., 2011. Real and hypothetical rewards. Judgment and Decision Making 6, 552–564.
124. Locey, M.L., Rachlin, H., 2012. COMMITMENT AND SELF-CONTROL IN A PRISONER's DILEMMA GAME. Journal of the Experimental Analysis of Behavior 98, 89–103. https://doi.org/10.1901/jeab.2012.98-89
125. Locey, M.L., Rachlin, H., 2011. Temporal Dynamics of Cooperation. Journal of Behavioral Decision Making 25, 257–263. https://doi.org/10.1002/bdm.729
126. Locey, M.L., Safin, V., Rachlin, H., 2012. SOCIAL DISCOUNTING AND THE PRISONER's DILEMMA GAME. Journal of the Experimental Analysis of Behavior 99, 85–97. https://doi.org/10.1002/jeab.3
127. Lönnqvist, J.-E., Walkowitz, G., Wichardt, P., Lindeman, M., Verkasalo, M., 2009. The moderating effect of conformism values on the relations between other personal values, social norms, moral obligation, and single altruistic behaviours. British Journal of Social Psychology 48, 525–546. https://doi.org/10.1348/014466608x377396
128. Lount, R.B., Zhong, C.-B., Sivanathan, N., Murnighan, J.K., 2008. Getting Off on the Wrong Foot: The Timing of a Breach and the Restoration of Trust. Personality and Social Psychology Bulletin 34, 1601–1612. https://doi.org/10.1177/0146167208324512
129. Lukinova, E., Myagkov, M., 2016. Impact of Short Social Training on Prosocial Behaviors: An fMRI Study. Frontiers in Systems Neuroscience 10. https://doi.org/10.3389/fnsys.2016.00060
130. Luo, Y., Zhang, S., Tao, R., Geng, H., 2016. The power of subliminal and supraliminal eye contact on social decision making: An individual-difference perspective. Consciousness and Cognition 40, 131–140. https://doi.org/10.1016/j.concog.2016.01.001
131. Maeda, K., Hashimoto, H., 2020. Time Pressure and In-group Favoritism in a Minimal Group Paradigm. Front. Psychol. 11. https://doi.org/10.3389/fpsyg.2020.603117
132. Martin, J.M., Juvina, I., Lebiere, C., Gonzalez, C., 2013. The effects of individual and context on aggression in repeated social interaction. Applied Ergonomics 44, 710–718. https://doi.org/10.1016/j.apergo.2012.04.014
133. Matsumoto, Y., Yamagishi, T., Li, Y., Kiyonari, T., 2016. Prosocial Behavior Increases with Age across Five Economic Games. PLOS ONE 11, e0158671. https://doi.org/10.1371/journal.pone.0158671
134. McClure, E.B., Parrish, J.M., Nelson, E.E., Easter, J., Thorne, J.F., Rilling, J.K., Ernst, M., Pine, D.S., 2007. Responses to Conflict and Cooperation in Adolescents with Anxiety and Mood Disorders. Journal of Abnormal Child Psychology 35, 567–577. https://doi.org/10.1007/s10802-007-9113-8
135. McClure, M.J., Bartz, J.A., Lydon, J.E., 2013. Uncovering and Overcoming Ambivalence: The Role of Chronic and Contextually Activated Attachment in Two-Person Social Dilemmas. Journal of Personality 81, 103–117. https://doi.org/10.1111/j.1467-6494.2012.00788.x
136. McClure-Tone, E.B., Nawa, N.E., Nelson, E.E., Detloff, A.M., Fromm, S.J., Pine, D.S., Ernst, M., 2011. Preliminary Findings: Neural Responses to Feedback Regarding Betrayal and Cooperation in Adolescent Anxiety Disorders. Developmental Neuropsychology 36, 453–472. https://doi.org/10.1080/87565641.2010.549876
137. Melamed, D., Simpson, B., Harrell, A., 2017. Prosocial Orientation Alters Network Dynamics and Fosters Cooperation. Scientific Reports 7. https://doi.org/10.1038/s41598-017-00265-x
138. Mengel, F., 2014. Computer Games and Prosocial Behaviour. PLoS ONE 9, e94099. https://doi.org/10.1371/journal.pone.0094099

139. Menshikov, I.S., Shklover, A.V., Babkina, T.S., Myagkov, M.G., 2017. From rationality to cooperativeness: The totally mixed Nash equilibrium in Markov strategies in the iterated Prisoner's Dilemma. PLOS ONE 12, e0180754. https://doi.org/10.1371/journal.pone.0180754
140. Mienaltowski, A., Wichman, A.L., 2020. Older and younger adults' interactions with friends and strangers in an iterated prisoner's dilemma. Neuropsychol Dev Cogn B Aging Neuropsychol Cogn 27, 153–172. https://doi.org/10.1080/13825585.2019.1598537
141. Mieth, L., Bell, R., Buchner, A., 2016a. Facial Likability and Smiling Enhance Cooperation, but Have No Direct Effect on Moralistic Punishment. Experimental Psychology 63, 263–277. https://doi.org/10.1027/1618-3169/a000338
142. Mieth, L., Bell, R., Buchner, A., 2016b. Cognitive Load Does Not Affect the Behavioral and Cognitive Foundations of Social Cooperation. Frontiers in Psychology 7. https://doi.org/10.3389/fpsyg.2016.01312
143. Mieth, L., Schaper, M.L., Kuhlmann, B.G., Bell, R., 2021. Memory and metamemory for social interactions: Evidence for a metamemory expectancy illusion. Memory & Cognition 49, 14–31. https://doi.org/10.3758/s13421-020-01071-z
144. Milinski, M., Wedekind, C., 1998. Working memory constrains human cooperation in the Prisoner's Dilemma. Proceedings of the National Academy of Sciences 95, 13755–13758. https://doi.org/10.1073/pnas.95.23.13755
145. Mokros, A., Menner, B., Eisenbarth, H., Alpers, G.W., Lange, K.W., Osterheider, M., 2008. Diminished cooperativeness of psychopaths in a prisoner's dilemma game yields higher rewards. Journal of Abnormal Psychology 117, 406–413. https://doi.org/10.1037/0021-843x.117.2.406
146. Neto, M.L., Antunes, M., Lopes, M., Ferreira, D., Rilling, J., Prata, D., 2020. Oxytocin and vasopressin modulation of prisoner's dilemma strategies. Journal of Psychopharmacology 34, 891–900. https://doi.org/10.1177/0269881120913145
147. Ng, G.T.T., Au, W.T., 2015. Expectation and cooperation in prisoner's dilemmas: The moderating role of game riskiness. Psychonomic Bulletin & Review 23, 353–360. https://doi.org/10.3758/s13423-015-0911-7
148. Pansini, R., Shi, L., Wang, R.-W., 2016. Women Tend to Defect in a Social Dilemma Game in Southwest China. PLOS ONE 11, e0166101. https://doi.org/10.1371/journal.pone.0166101
149. Papageorgiou, C., Karanasiou, I.S., Tsianaka, E.I., Kyprianou, M., Papadimitriou, G.N., Uzunoglu, N.K., 2013. Motive related positivity: Decision-making during a prisoners' dilemma task. Journal of Integrative Neuroscience 12, 183–199. https://doi.org/10.1142/s0219635213500106
150. Peshkovskaya, A.G., Babkina, T.S., Myagkov, M.G., Kulikov, I.A., Ekshova, K.V., Harriff, K., 2017. The socialization effect on decision making in the Prisoner's Dilemma game: An eye-tracking study. PLOS ONE 12, e0175492. https://doi.org/10.1371/journal.pone.0175492
151. Pfeiffer, T., Tran, L., Krumme, C., Rand, D.G., 2012. The value of reputation. Journal of The Royal Society Interface 9, 2791–2797. https://doi.org/10.1098/rsif.2012.0332
152. Pinel, E.C., Long, A.E., Huneke, M., 2015. In the Blink of an I: On Delayed but Identical Subjective Reactions and Their Effect on Self-Interested Behavior. The Journal of Social Psychology 155, 605–616. https://doi.org/10.1080/00224545.2015.1038496
153. Podd, M.H., Marcia, J.E., Rubin, B.M., 1970. The Effects of Ego Identity and Partner Perception on a Prisoner's Dilemma Game. The Journal of Social Psychology 82, 117–126. https://doi.org/10.1080/00224545.1970.9919936
154. Press, W.H., Dyson, F.J., 2012. Iterated Prisoner's Dilemma contains strategies that dominate any evolutionary opponent. Proceedings of the National Academy of Sciences 109, 10409–10413. https://doi.org/10.1073/pnas.1206569109
155. Prétôt, L., Gonzalez, G., McAuliffe, K., 2020. Children avoid inefficient but fair partners in a cooperative game. Sci Rep 10, 10511. https://doi.org/10.1038/s41598-020-65452-9
156. Prétôt, L., McAuliffe, K., 2020. Does nonbinding commitment promote children's cooperation in a social dilemma? J Exp Child Psychol 200, 104947. https://doi.org/10.1016/j.jecp.2020.104947
157. Pulcu, E., Thomas, E.J., Trotter, P.D., McFarquhar, M., Juhasz, G., Sahakian, B.J., Deakin, J.F.W., Anderson, I.M., Zahn, R., Elliott, R., 2014. Social-economical decision making in current and remitted major depression. Psychological Medicine 45, 1301–1313. https://doi.org/10.1017/s0033291714002414
158. Rapoport, A., 1967. A note on the index of cooperation for prisoner's dilemma. Journal of Conflict Resolution 11, 100–103.
159. Rapoport, A., Mowshowitz, A., 1966. Experimental studies of stochastic models for the Prisoner's dilemma. Behavioral Science 11, 444–458. https://doi.org/10.1002/bs.3830110604
160. Rawolle, M., Schultheiss, O.C., Strasser, A., Kehr, H.M., 2016. The Motivating Power of Visionary Images: Effects on Motivation, Affect, and Behavior. Journal of Personality 85, 769–781. https://doi.org/10.1111/jopy.12285
161. Reimers, L., Diekhof, E.K., 2015. Testosterone is associated with cooperation during intergroup competition by enhancing parochial altruism. Frontiers in Neuroscience 9. https://doi.org/10.3389/fnins.2015.00183
162. Rilling, J.K., Chen, Xiangchuan, Chen, Xu, Haroon, E., 2018. Intranasal oxytocin modulates neural functional connectivity during human social interaction. American Journal of Primatology 80, e22740. https://doi.org/10.1002/ajp.22740
163. Rilling, J.K., DeMarco, A.C., Hackett, P.D., Chen, X., Gautam, P., Stair, S., Haroon, E., Thompson, R., Ditzen, B., Patel, R., Pagnoni, G., 2014. Sex differences in the neural and behavioral response to intranasal oxytocin and vasopressin during human social interaction. Psychoneuroendocrinology 39, 237–248. https://doi.org/10.1016/j.psyneuen.2013.09.022
164. Rilling, J.K., DeMarco, A.C., Hackett, P.D., Thompson, R., Ditzen, B., Patel, R., Pagnoni, G., 2012. Effects of intranasal oxytocin and vasopressin on cooperative behavior and associated brain activity in men. Psychoneuroendocrinology 37, 447–461. https://doi.org/10.1016/j.psyneuen.2011.07.013
165. Rilling, J.K., Glenn, A.L., Jairam, M.R., Pagnoni, G., Goldsmith, D.R., Elfenbein, H.A., Lilienfeld, S.O., 2007. Neural Correlates of Social Cooperation and Non-Cooperation as a Function of Psychopathy. Biological Psychiatry 61, 1260–1271. https://doi.org/10.1016/j.biopsych.2006.07.021
166. Rilling, J.K., Gutman, D.A., Zeh, T.R., Pagnoni, G., Berns, G.S., Kilts, C.D., 2002. A Neural Basis for Social Cooperation. Neuron 35, 395–405. https://doi.org/10.1016/s0896-6273(02)00755-9
167. Rilling, J.K., Sanfey, A.G., Aronson, J.A., Nystrom, L.E., Cohen, J.D., 2004a. The neural correlates of theory of mind within interpersonal interactions. NeuroImage 22, 1694–1703. https://doi.org/10.1016/j.neuroimage.2004.04.015
168. Rilling, J.K., Sanfey, A.G., Aronson, J.A., Nystrom, L.E., Cohen, J.D., 2004b. Opposing BOLD responses to reciprocated and unreciprocated altruism in putative reward pathways. NeuroReport 15, 2539–2243. https://doi.org/10.1097/00001756-200411150-00022
169. Rodebaugh, T.L., Klein, S.R., Yarkoni, T., Langer, J.K., 2011. Measuring social anxiety related interpersonal constraint with the flexible iterated prisoner's dilemma. Journal of Anxiety Disorders 25, 427–436. https://doi.org/10.1016/j.janxdis.2010.11.006
170. Rodebaugh, T.L., Shumaker, E.A., Levinson, C.A., Fernandez, K.C., Langer, J.K., Lim, M.H., Yarkoni, T., 2013. Interpersonal constraint conferred by generalized social anxiety disorder is evident on a behavioral economics task. Journal of Abnormal Psychology 122, 39–44. https://doi.org/10.1037/a0030975
171. Rodriguez-Ruiz, C., Muñoz-Reyes, J.A., Iglesias-Julios, M., Sanchez-Pages, S., Turiegano, E., 2019. Sex Affects the Relationship Between Third Party Punishment and Cooperation. Sci Rep 9, 4288. https://doi.org/10.1038/s41598-019-40909-8
172. Rostovtseva, V.V., Weissing, F.J., Mezentseva, A.A., Butovskaya, M.L., 2020. Sex differences in cooperativeness—An experiment with Buryats in Southern Siberia. PLOS ONE 15, e0239129. https://doi.org/10.1371/journal.pone.0239129
173. Rotenberg, K.J., Macdonald, K.J., King, E.V., 2004. The Relationship Between Loneliness and Interpersonal Trust During Middle Childhood. The Journal of Genetic Psychology 165, 233–249. https://doi.org/10.3200/gntp.165.3.233-249
174. Safin, V., Arfer, K.B., Rachlin, H., 2015. Reciprocation and altruism in social cooperation. Behavioural Processes 116, 12–16. https://doi.org/10.1016/j.beproc.2015.04.009
175. Safin, V., Locey, M.L., Rachlin, H., 2013. Valuing rewards to others in a prisoner's dilemma game. Behavioural Processes 99, 145–149. https://doi.org/10.1016/j.beproc.2013.07.008
176. Sakaiya, S., Shiraito, Y., Kato, J., Ide, H., Okada, K., Takano, K., Kansaku, K., 2013. Neural correlate of human reciprocity in social interactions. Frontiers in Neuroscience 7. https://doi.org/10.3389/fnins.2013.00239
177. Samu, F., Számadó, S., Takács, K., 2020. Scarce and directly beneficial reputations support cooperation. Sci Rep 10, 11486. https://doi.org/10.1038/s41598-020-68123-x
178. Saunders, K.E.A., Goodwin, G.M., Rogers, R.D., 2015. Borderline personality disorder, but not euthymic bipolar disorder, is associated with a failure to sustain reciprocal cooperative behaviour: implications for spectrum models of mood disorders. Psychological Medicine 45, 1591–1600. https://doi.org/10.1017/s0033291714002475
179. Savulich, G., Jeanes, H., Rossides, N., Kaur, S., Zacharia, A., Robbins, T.W., Sahakian, B.J., 2018. Moral Emotions and Social Economic Games in Paranoia. Frontiers in Psychiatry 9. https://doi.org/10.3389/fpsyt.2018.00615
180. Schaper, M.L., Mieth, L., Bell, R., 2019. Adaptive memory: Source memory is positively associated with adaptive social decision making. Cognition 186, 7–14. https://doi.org/10.1016/j.cognition.2019.01.014
181. Schneider-Hassloff, H., Straube, B., Jansen, A., Nuscheler, B., Wemken, G., Witt, S.H., Rietschel, M., Kircher, T., 2016. Oxytocin receptor polymorphism and childhood social experiences shape adult personality, brain structure and neural correlates of mentalizing. NeuroImage 134, 671–684. https://doi.org/10.1016/j.neuroimage.2016.04.009
182. Schneider-Hassloff, H., Straube, B., Nuscheler, B., Wemken, G., Kircher, T., 2015. Adult attachment style modulates neural responses in a mentalizing task. Neuroscience 303, 462–473. https://doi.org/10.1016/j.neuroscience.2015.06.062
183. Sermat, V., 1967. The possibility of influencing the other's behaviour and co-operation: Chicken versus prisoner's dilemma. Canadian Journal of Psychology/Revue canadienne de psychologie 21, 204–219. https://doi.org/10.1037/h0082982
184. Shafir, E., Tversky, A., 1992. Thinking through uncertainty: Nonconsequential reasoning and choice. Cognitive Psychology 24, 449–474. https://doi.org/10.1016/0010-0285(92)90015-t
185. Sheldon, K.M., Sheldon, M.S., Osbaldiston, R., 2000. Prosocial values and group assortation. Human Nature 11, 387–404. https://doi.org/10.1007/s12110-000-1009-z
186. Singer, T., Kiebel, S.J., Winston, J.S., Dolan, R.J., Frith, C.D., 2004. Brain Responses to the Acquired Moral Status of Faces. Neuron 41, 653–662. https://doi.org/10.1016/s0896-6273(04)00014-5
187. Smith, T.W., Brehm, S.S., 1981. Person perception and the Type A coronary-prone behavior pattern. Journal of Personality and Social Psychology 40, 1137–1149. https://doi.org/10.1037/0022-3514.40.6.1137
188. Sorgi, K.M., Wout, M. van 't, 2016. The influence of cooperation and defection on social decision making in depression: A study of the iterated Prisoner's Dilemma Game. Psychiatry Research 246, 512–519. https://doi.org/10.1016/j.psychres.2016.10.025
189. Soutschek, A., Sauter, M., Schubert, T., 2015. The Importance of the Lateral Prefrontal Cortex for Strategic Decision Making in the Prisoner's Dilemma. Cognitive, Affective, & Behavioral Neuroscience 15, 854–860. https://doi.org/10.3758/s13415-015-0372-5
190. Stevens, J.R., Volstorf, J., Schooler, L.J., Rieskamp, J., 2011. Forgetting Constrains the Emergence of Cooperative Decision Strategies. Frontiers in Psychology 1. https://doi.org/10.3389/fpsyg.2010.00235
191. Storey, S., Workman, L., 2013. The effects of temperature priming on cooperation in the iterated prisoner's dilemma. Evolutionary Psychology : An International Journal of Evolutionary Approaches to Psychology and Behavior 11, 52–67.
192. Sun, P., Zheng, L., Li, L., Guo, X., Zhang, W., Zheng, Y., 2016. The Neural Responses to Social Cooperation in Gain and Loss Context. PLOS ONE 11, e0160503. https://doi.org/10.1371/journal.pone.0160503
193. Suzuki, S., Niki, K., Fujisaki, S., Akiyama, E., 2010. Neural basis of conditional cooperation. Social Cognitive and Affective Neuroscience 6, 338–347. https://doi.org/10.1093/scan/nsq042
194. Swingle, P.G., 1968. Effects of prior exposure to cooperative or competitive treatment upon subject's responding in the Prisoner's Dilemma. Journal of Personality and Social Psychology 10, 44–52. https://doi.org/10.1037/h0026311
195. Swingle, P.G., Coady, H., 1967. Effects of the partner's abrupt strategy change upon subject's responding in the Prisoner's Dilemma. Journal of Personality and Social Psychology 5, 357–363. https://doi.org/10.1037/h0024306
196. Tabak, B.A., McCullough, M.E., Carver, C.S., Pedersen, E.J., Cuccaro, M.L., 2013. Variation in oxytocin receptor gene (OXTR) polymorphisms is associated with emotional and behavioral reactions to betrayal. Social Cognitive and Affective Neuroscience 9, 810–816. https://doi.org/10.1093/scan/nst042
197. Tabak, B.A., McCullough, M.E., Luna, L.R., Bono, G., Berry, J.W., 2012. Conciliatory Gestures Facilitate Forgiveness and Feelings of Friendship by Making Transgressors Appear More Agreeable. Journal of Personality 80, 503–536. https://doi.org/10.1111/j.1467-6494.2011.00728.x
198. Taheri, M., Rotshtein, P., Beierholm, U., 2018. The effect of attachment and environmental manipulations on cooperative behavior in the prisoner's dilemma game. PLOS ONE 13, e0205730. https://doi.org/10.1371/journal.pone.0205730
199. Takemura, K., Yuki, M., 2007. Are Japanese groups more competitive than Japanese individuals? A cross-cultural validation of the interindividual–intergroup discontinuity effect. International Journal of Psychology 42, 27–35. https://doi.org/10.1080/00207590500411534
200. Tayama, M., Tateno, M., Park, T.W., Ukai, W., Hashimoto, E., Saito, T., 2012. The Study of Cognitive Characteristics in Asperger's Disorder by Using a Modified Prisoner's Dilemma Game with a Variable Payoff Matrix. PLoS ONE 7, e48794. https://doi.org/10.1371/journal.pone.0048794
201. Tedeschi, J., Lesnick, S., Gahagan, J., 1968. Feedback and "washout" effects in the Prisoner's Dilemma game. Journal of Personality and Social Psychology 10, 31–34. https://doi.org/10.1037/h0026312
202. Testori, M., Harris, T.O.A., Hoyle, R.B., Eisenbarth, H., 2019. The effect of psychopathy on cooperative strategies in an iterated Prisoner's Dilemma experiment with emotional feedback. Scientific Reports 9. https://doi.org/10.1038/s41598-019-38796-0
203. Thompson, K., King, K., Nahmias, E., Fani, N., Kvaran, T., Tone, E.B., Turner, J.A., 2019. Social Feedback Modulates Neural Response Associated With Cognitive Bias in Individuals Expressing Anxious Symptoms. Chronic Stress 3, 2470547019848648. https://doi.org/10.1177/2470547019848648
204. Viola, T.W., Niederauer, J.P.O., Kluwe-Schiavon, B., Sanvicente-Vieira, B., Grassi-Oliveira, R., 2019. Cocaine use disorder in females is associated with altered social decision-making: a study with the prisoner's dilemma and the ultimatum game. BMC Psychiatry 19, 211. https://doi.org/10.1186/s12888-019-2198-0
205. Vives, M.-L., FeldmanHall, O., 2018. Tolerance to ambiguous uncertainty predicts prosocial behavior. Nature Communications 9. https://doi.org/10.1038/s41467-018-04631-9
206. Volstorf, J., Rieskamp, J., Stevens, J.R., 2011. The Good, the Bad, and the Rare: Memory for Partners in Social Interactions. PLoS ONE 6, e18945. https://doi.org/10.1371/journal.pone.0018945
207. Wang, J., Suri, S., Watts, D.J., 2012. Cooperation and assortativity with dynamic partner updating. Proceedings of the National Academy of Sciences 109, 14363–14368. https://doi.org/10.1073/pnas.1120867109
208. Wang, Z., Jusup, M., Shi, L., Lee, J.-H., Iwasa, Y., Boccaletti, S., 2018. Exploiting a cognitive bias promotes cooperation in social dilemma experiments. Nature Communications 9. https://doi.org/10.1038/s41467-018-05259-5
209. Wang, Z., Zhou, Y., Lien, J.W., Zheng, J., Xu, B., 2016. Extortion can outperform generosity in the iterated prisoner's dilemma. Nature Communications 7. https://doi.org/10.1038/ncomms11125
210. Wedekind, C., Milinski, M., 1996. Human cooperation in the simultaneous and the alternating Prisoner's Dilemma: Pavlov versus Generous Tit-for-Tat. Proceedings of the National Academy of Sciences 93, 2686–2689. https://doi.org/10.1073/pnas.93.7.2686
211. Weisel, O., Böhm, R., 2015. "Ingroup love" and "outgroup hate" in intergroup conflict between natural groups. Journal of Experimental Social Psychology 60, 110–120. https://doi.org/10.1016/j.jesp.2015.04.008
212. West, S.A., Gardner, A., Shuker, D.M., Reynolds, T., Burton-Chellow, M., Sykes, E.M., Guinnee, M.A., Griffin, A.S., 2006. Cooperation and the Scale of Competition in Humans. Current Biology 16, 1103–1106. https://doi.org/10.1016/j.cub.2006.03.069
213. Wildschut, T., Horen, F. van, Hart, C., 2015. Accountability Accentuates Interindividual-Intergroup Discontinuity by Enforcing Parochialism. Frontiers in Psychology 6. https://doi.org/10.3389/fpsyg.2015.01789
214. Wildschut, T., Insko, C.A., Gaertner, L., 2002. Intragroup social influence and intergroup competition. Journal of Personality and Social Psychology 82, 975–992. https://doi.org/10.1037/0022-3514.82.6.975
215. Wolf, S.T., Insko, C.A., Kirchner, J.L., Wildschut, T., 2008. Interindividual-intergroup discontinuity in the domain of correspondent outcomes: The roles of relativistic concern, perceived categorization, and the doctrine of mutual assured destruction. Journal of Personality and Social Psychology 94, 479–494. https://doi.org/10.1037/0022-3514.94.3.479
216. Wong, R.Y.M., Hong, Y.Y., 2005. Dynamic influences of culture on cooperation in the prisoner's dilemma. Psychological Science 16, 429–434. https://doi.org/10.1111/j.0956-7976.2005.01552.x
217. Wood, R.M., Rilling, J.K., Sanfey, A.G., Bhagwagar, Z., Rogers, R.D., 2006. Effects of Tryptophan Depletion on the Performance of an Iterated Prisoner's Dilemma Game in Healthy Adults. Neuropsychopharmacology 31, 1075–1084. https://doi.org/10.1038/sj.npp.1300932
218. Yamagishi, T., Horita, Y., Mifune, N., Hashimoto, H., Li, Y., Shinada, M., Miura, A., Inukai, K., Takagishi, H., Simunovic, D., 2012. Rejection of unfair offers in the ultimatum game is no evidence of strong reciprocity. Proceedings of the National Academy of Sciences 109, 20364–20368. https://doi.org/10.1073/pnas.1212126109
219. Yamagishi, T., Li, Y., Takagishi, H., Matsumoto, Y., Kiyonari, T., 2014. In Search of Homo economicus. Psychological Science 25, 1699–1711. https://doi.org/10.1177/0956797614538065
220. Yang, C.-L., Yue, C.-S.J., 2019. Cooperation in an Assortative Matching Prisoners Dilemma Experiment with Pro-Social Dummies. Sci Rep 9, 13609. https://doi.org/10.1038/s41598-019-50083-6
221. Yang, Q., Wu, X., Zhou, X., Mead, N.L., Vohs, K.D., Baumeister, R.F., 2013. Diverging effects of clean versus dirty money on attitudes, values, and interpersonal behavior. Journal of Personality and Social Psychology 104, 473–489. https://doi.org/10.1037/a0030596
222. Yao, Z., Yu, R., 2016. The Spreading of Social Energy: How Exposure to Positive and Negative Social News Affects Behavior. PLOS ONE 11, e0156062. https://doi.org/10.1371/journal.pone.0156062
223. Yi, R., Buchhalter, A.R., Gatchalian, K.M., Bickel, W.K., 2007. The relationship between temporal discounting and the prisoner's dilemma game in intranasal abusers of prescription opioids. Drug and Alcohol Dependence 87, 94–97. https://doi.org/10.1016/j.drugalcdep.2006.07.007
224. Yi, R., Johnson, M.W., Bickel, W.K., 2005. Relationship between cooperation in an iterated prisoner's dilemma game and the discounting of hypothetical outcomes. Learning & Behavior 33, 324–336. https://doi.org/10.3758/bf03192861
225. Yi, R., Rachlin, H., 2004. CONTINGENCIES OF REINFORCEMENT IN A FIVE-PERSON PRISONER's DILEMMA. Journal of the Experimental Analysis of Behavior 82, 161–176. https://doi.org/10.1901/jeab.2004.82-161
226. Zhang, D., Lin, Y., Jing, Y., Feng, C., Gu, R., 2019. The Dynamics of Belief Updating in Human Cooperation: Findings from inter-brain ERP hyperscanning. Neuroimage 198, 1–12. https://doi.org/10.1016/j.neuroimage.2019.05.029
227. Zhang, M., Ding, K., Jia, H., Yu, D., 2018. Brain-to-brain synchronization of the expectation of cooperation behavior: A fNIRS hyperscanning study, in: 2018 40th Annual International Conference of the IEEE Engineering in Medicine and Biology Society (EMBC). IEEE. https://doi.org/10.1109/embc.2018.8512315
228. Zhang, M., Jia, H., Zheng, M., 2020. Interbrain synchrony in the expectation of cooperation behavior: A hyperscanning study using functional near-infrared spectroscopy. Frontiers in psychology 11.
